# Pulp–Dentin Regeneration via Cell Homing: Current Evidence and Perspectives on Cell-Free Regenerative Endodontic Therapy

**DOI:** 10.3390/medicina62020375

**Published:** 2026-02-13

**Authors:** Michele Beco, Francesca Di Pasquale, Chiara Valenti, Paolo Betti, Gian Luca Mascolo, Lorella Marinucci, Stefano Eramo, Stefano Pagano

**Affiliations:** 1 Department of Medicine and Surgery, Faculty of Dentistry, University of Perugia, Sant’Andrea delle Fratte, 06156 Perugia, Italy; michele.beco@studenti.unipg.it (M.B.); francesca.dipasquale@outlook.it (F.D.P.); paolo.b1998@gmail.com (P.B.); gianluca.mascolo@unipg.it (G.L.M.); stefano.eramo@unipg.it (S.E.); stefano.pagano@unipg.it (S.P.); 2 CISAS “Giuseppe Colombo”, University of Padua, Via Venezia, 1, 35131 Padua, Italy; 3 Department of Medicine and Surgery, Section of Human Anatomy, University of Perugia, Sant’Andrea delle Fratte, 06156 Perugia, Italy; lorella.marinucci@unipg.it

**Keywords:** cell homing, regenerative endodontic therapy, pulp–dentin regeneration, pulp revascularization, scaffolds, stem cell migration

## Abstract

*Background and Objectives*: The regeneration of the pulp–dentin complex represents an alternative to conventional root canal treatment, aiming to preserve tooth biology and function. Cell-free regenerative endodontic therapy (CF-RET) exploits endogenous stem cells from the periapical region without ex vivo cell manipulation. Despite growing interest, the biological mechanisms, clinical indications, and predictability of CF-RET remain not clearly defined. This structured narrative review aimed to update a previous review by analyzing recent human studies on CF-RET. *Materials and Methods*: This review was conducted using the PRISMA 2020 guidelines to guide transparent reporting of the literature search and study selection process and was registered in PROSPERO (CRD420251075131). In vitro and in vivo human studies published between January 2017 and December 2024 investigating CF-RET were included, while studies involving cell transplantation, non-human models, case reports, and reviews were excluded. Study selection, data extraction, and quality assessment using the QuADS tool were performed, and the evidence was synthesized using a qualitative narrative approach. *Results*: Sixty-four studies were included. In vitro studies reported favorable effects of growth factors, exosomes, and biomimetic scaffolds on stem cell viability, migration, proliferation, odontogenic differentiation, and angiogenesis, while neurogenic differentiation was less consistently investigated. Scaffold composition, microstructure, and rheological properties were also considered. In vivo studies mainly focused on immature teeth with incomplete root development and demonstrated positive clinical and radiographic outcomes, including root development and canal diameter reduction. *Conclusions*: The current evidence supports the biological potential of CF-RET as a regenerative approach; however, substantial heterogeneity, the limited number of clinical studies and the absence of standardized protocols preclude definitive conclusions, highlighting the need for further well-designed translational and clinical investigations considering clinical applicability.

## 1. Introduction

The regeneration of the pulp–dentin complex is an ambitious technique aimed at regenerating pulp tissue and has been extensively investigated to achieve predictable and clinically applicable outcomes. Chemical or mechanical agents, such as caries, exposed dentin, dental trauma, recent dental treatments, or inadequate restorations, can lead to pulpitis, an inflammation of the dental pulp. Clinically, pulpitis is distinguished as reversible or irreversible based on the reversibility of the inflammatory process.

Irreversible pulpitis necessitates treatment to remove the affected pulp and its replacement with an inert material. Root canal treatment (RCT), despite adherence to the standard procedure, is associated with a percentage of failures and adverse events, attributed to microbial factors stemming from persistent or secondary root canal infections, extra-radicular infections originating from the periapical lesion caused by pulpal pathology, or non-microbial extrinsic factors [1].

Due to the loss of nerve fibers within the pulp following RCT, the patient cannot perceive thermal, electrical, or osmotic stimuli as if the tooth were vital and thus loses the ability to sense potential active pathological conditions. The capacity for the generation of secondary or tertiary dentin is also lost, as odontoblasts are removed during RCT, and endodontically treated teeth are more vulnerable to masticatory forces [2]. Dental caries and traumatic injuries can also affect immature teeth and, similarly to teeth with complete root formation, can lead to pulpal necrosis [3]. In immature teeth, the removal of odontoblasts prevents complete root development. Teeth treated with RCT before root development is complete remain with shorter roots and larger apical foramina. Currently, pulp revascularization is employed to facilitate the complete development of the tooth roots.

The pulp revascularization technique is based on, once the root canal has been disinfected with a combination of antibiotics or calcium hydroxide, inducing bleeding from the apical papilla with a file to allow the formation of a blood clot within the root canal. This process facilitates the migration of stem cells that can differentiate and produce dentin and ultimately leading to the completion of root development. Stem cells, present in large numbers in the periapical tissue, can differentiate into various cell types: osteo/odontogenic, neurogenic, and other lineages, such as chondrocytes and even adipocytes [4]. It has been hypothesized that the blood clot acts as a scaffold that facilitates the migration and engraftment of stem cells [5]. The blood clot is sealed within the root canal using mineral trioxide aggregate (MTA) or other bioactive endodontic cements [6]. However, the formation of this blood clot does not equate to the regeneration of an organized pulp tissue within which blood vessels and nerves. A histological examination with hematoxylin and eosin staining reveals the presence of connective tissue, fibroblasts, and blood vessels with few lymphocytes and an absence of odontoblasts [7]. Dentin development is arrested, and the thickness of the walls increases due to the formation of an ectopic, irregular cementum-like tissue on the surface of the root canal walls. In some specimens, a soft tissue, such as the periodontal ligament, where Sharpey’s fibers can also be identified, forms along this ectopic cementum. A regular layer of odontoblasts rarely forms, while bone bridges may develop within the canal [8]. A histological analysis conducted in one study revealed that only 53% of the roots in the four experimental groups (with previously infected canals) exhibited new hard tissue deposition on the internal root canal walls. Therefore, revascularization does not equate to regeneration but, rather, to tissue repair [9].

Currently, the regenerative approaches targeting the pulp–dentin complex can be broadly distinguished according to the origin of the cells involved. A recent classification [10] differentiates these strategies into cell-based regenerative endodontic therapy (CB-RET) and cell-free regenerative endodontic therapy (CF-RET). In CB-RET, stem cells derived from the host (autologous) or donors (allogeneic) are transplanted into the root canal following ex vivo expansion, with the aim of directly repopulating the pulp space and achieving true tissue regeneration. Despite its theoretical advantages, this approach presents significant limitations, including unpredictable cell availability, a risk of contamination, immune rejection, high costs, and regulatory barriers associated with ex vivo cell manipulation [11].

In contrast, CF-RET relies on the recruitment of endogenous stem and progenitor cells, primarily from periapical tissues, through cell-homing mechanisms. This strategy employs intracanal scaffolds, bioactive molecules, and/or endogenous growth factor release to stimulate cell migration, proliferation, and differentiation within the disinfected root canal, without the need for cell transplantation. CF-RET is, therefore, conceptually distinct from CB-RET and is considered more clinically feasible [10].

Conventional revascularization procedures, although often grouped under the umbrella of regenerative endodontics, differ fundamentally in their intended biological mechanism and outcome. These techniques primarily aim to induce bleeding into the root canal to promote tissue ingrowth and resolution of infection, frequently resulting in reparative tissues such as connective tissue, cementum-like tissue, or bone, rather than the consistent regeneration of a functional pulp–dentin complex.

Despite the clinical appeal of CF-RET, important uncertainties remain regarding the specific cell populations involved in tissue formation and whether stem cell recruitment originates exclusively from the apical region [12]. Moreover, current evidence does not demonstrate significant differences between CF-RET and CB-RET in terms of apical bone healing and findings related to tooth maturation remain conflicting. While CB-RET has been associated with more complete pulp-like tissue formation, CF-RET outcomes have occasionally shown altered or non-native tissue organization. However, the literature currently provides limited material on the evaluation of these aspects, which necessitate a histological approach [13]. Notably, none of the previously published works specifically focus on CF-RET by collecting both in vivo and in vitro studies but, rather, exclusively concentrate on individual aspects mentioned above, offering a simple comparison between the two studied regeneration methods [11].

The aim of the present structured narrative review was to update and supplement a 2017 work by Eramo et al. with the most recent in vitro and in vivo studies on CF-RET [14]. This review intends to provide an overview of the CF-RET method, stem cells, growth factors, and materials used for scaffolds in pulp–dentin regeneration, analyzing the advancements in in situ pulp revascularization, evaluating whether the fundamental principles guiding research remain the same, the mode of stem cell migration into the root canal, the conditions for migration, proliferation, and attachment, and the various growth factors that promote this process and support the potential complete regeneration of the pulp.

## 2. Materials and Methods

**Figure 1:**
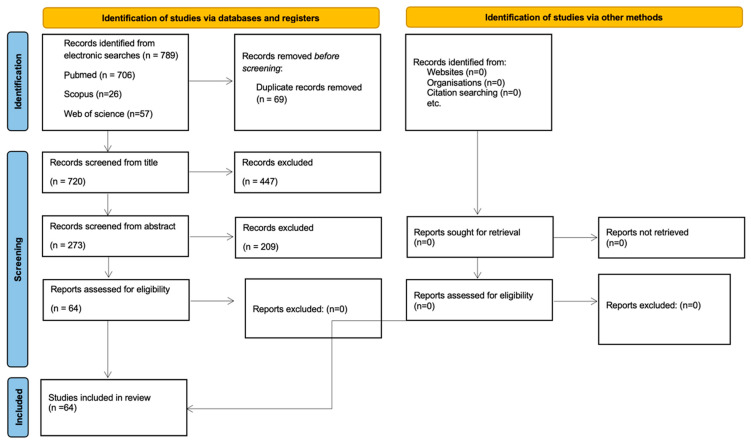
Flow diagram of the literature search and study selection process.

This structured narrative review was conducted using the preferred reporting items for systematic review and meta-analysis (PRISMA 2020 statement) as a reporting framework to ensure the transparency and reproducibility of the literature search and study selection process [15]. A PRISMA flow diagram (Figure 1) was used to report the study inclusion process. The review protocol was registered in the PROSPERO database (CRD420251075131) to enhance methodological transparency and avoid unnecessary duplication, despite the narrative nature of the evidence synthesis. The Population, Inclusion, Comparison, Outcome, and Study design (PICOS) format was used to structure the research question and eligibility criteria (Table 1), based on the following question: “What is the current state of knowledge regarding the effects of CF-RET or cell-homing technique on human cell populations?”, without the intent of performing quantitative synthesis. This review is an update to a previous study [14].

The search for articles was conducted in MEDLINE (via PubMed) using the following combination of terms: ((((“Regenerative Endodontics” [Mesh] OR “Pulp regeneration” [All Fields]) AND (“Cell-Free Regenerative Endodontic Therapy” [All Fields] OR “CF-RET” [All Fields]) OR “Cell homing” [All Fields] OR “cell-free” [ All Fields] OR “endogenous stem cells” [All Fields] OR “stem cell recruitment” [All Fields])) AND (“scaffold” [All Fields] OR “growth factor *” [All Fields] OR “PRF” [All Fields] OR “PRP” [All Fields] OR “CGF” [All Fields])) NOT (Systematic Review [Publication Type] OR Review [Publication Type] OR Meta-Analysis [Publication Type] OR Congress [Publication Type] OR Editorial [Publication Type] OR Case Reports [Publication Type] OR Clinical Conference [Publication Type] OR Comment [Publication Type] OR Consensus Development Conference [Publication Type])) AND ((“1 January 2017” [Date—Publication]: “31 December 2024” [Date—Publication]))). The terms were adapted for the Scopus and Web of Science databases. Only articles in English or Italian were considered.

Studies published between 2017 and 2024 on CF-RET, in vitro and in vivo, considering the use of stem cells, possibly associated with scaffolds, cytokines, or additional treatments essential for completing pulp regeneration, were included.

Exclusion criteria: Studies on non-human cell populations, case reports, review articles, editorials, opinions, surveys, guidelines, conferences, and commentary articles. Studies without original and/or actual data, studies on cell transplantation, on therapies for vital pulp, on materials with biological effects on dentine or stem cells but without reference to pulp regeneration via cell homing, or on regeneration of tissues other than pulp or dentine. Studies with no full text available.

Screening was performed in three stages, based on the title and abstract, and then by a reading of the full text of the articles that potentially met the inclusion criteria. Electronic searches were followed by a manual search of the reference lists of included articles. At each stage, two reviewers (MB and SE) independently assessed each article. The PRISMA flow diagram (Figure 1) was used to report the studies included and excluded with justification.

The titles of all articles that were initially retrieved were screened according to eligibility criteria, and duplicates were removed. Two independent reviewers assessed the titles, and those that were not relevant were excluded. Articles compatible with the inclusion criteria were selected for further review, and abstracts were analyzed. The full texts of potentially eligible studies were reviewed against the inclusion/exclusion criteria independently by the reviewers, and any disagreements were solved through discussion or consultation with the other authors (SP and CV).

Scientific and technical information was collected in two evidence tables using Microsoft Office Excel, including in vitro and in vivo studies, with information on: the author(s) and year of publication, the study population (cell lines or patients), the CF-RET intervention, biological effects, the assay used and the conditions, and the principal findings. For data analysis, a narrative approach was adopted.

Two independent reviewers (MB and FDP) carried out the quality assessment of the included studies using a Quality Assessment Tool with diverse studies (QuADS) [16]. The QuADS tool was used to describe the methodological quality and reporting completeness of the included studies across different designs. Quality scores were not used to weight, exclude, or stratify studies, in line with the narrative nature of the evidence synthesis. This evaluation tool examines the methodological quality of the included studies and the extent to which a study addressed the possibility of bias in its design. The QuADS tool considered details on the rationale and aim, subjects and setting, study design, sampling and recruitment, data collection, exposure measurements, analysis methods selected, stake-holder involvements and limitations. These 13 evaluation criteria were rated on a scale from 0 to 3 (0: absence of the element; 1: very limited presence of the element; 2: moderate presence of the element; and 3: complete and adequate presence of the element).

## 3. Results

The flow diagram of screened studies (Figure 1) shows a total of 789 potentially eligible studies following the electronic search. Reviewer agreement and duplicate removal led to the elimination of 69 articles; title screening was completed on 720 studies, resulting in 447 non-eligible studies being excluded at this stage. Abstract screening was completed using 273 studies, with 64 progressing to full-text review. Finally, 64 studies were included in the full data analysis, and 0 studies were excluded after full-text reading [17,18,19,20,21,22,23,24,25,26,27,28,29,30,31,32,33,34,35,36,37,38,39,40,41,42,43,44,45,46,47,48,49,50,51,52,53,54,55,56,57,58,59,60,61,62,63,64,65,66,67,68,69,70,71,72,73,74,75,76,77,78]. One study was included in both the in vitro studies section and the in vivo studies section [9].

### 3.1. In Vitro Studies

Sixty papers were included in this narrative review, primarily investigating the behavior of dental stem cells SCAPs or DPSCs by supplementing the culture with specific molecules acting as growth factors to identify readily available and clinically applicable molecules that can enhance cellular viability. The most consistently evaluated outcomes included cell viability, migration, mineralization, and osteogenic/osteoblastic differentiation, all of which are considered fundamental parameters for successful pulp regeneration. In addition to determining the optimal concentration to elicit the desired effects, the studies investigating the role of growth factors have also aimed to find a simple and safe method for their activation, as well as a secure and clinically reliable delivery system [31,57,66]. At the concentrations used, all studies demonstrate the cellular differentiation of the stem cells, which is essential for the regeneration process [17,33,36,42,72], and the induction of new angiogenesis, which could facilitate the vascularization of the pulpal tissue [50,51,52,63,67,70,73].

Neurogenic differentiation, which is critical for establishing new innervation in the neo-formed pulpal tissue, was less frequently addressed and was reported in only three studies employing specific scaffold formulations [61,70,73]. Nevertheless, all scaffold-based approaches demonstrated satisfactory biocompatibility. Conversely, several studies have investigated the molecular mechanism of action through gene regulation to either activate or suppress specific genes responsible for cellular differentiation with the aim of elucidating how growth factors influence the regenerative process [26,34,41,46,60,63]. In addition, increasing attention has been directed toward stem cell–derived exosomes, which consistently enhanced cell viability, proliferation, migration, angiogenesis, and odontogenic differentiation [21,27,61,64,67,74,79,80,81]. Cell migration rate emerged as a recurring functional parameter that is fundamental because it plays a homeostatic role in tissue maintenance and is crucial for the regeneration of damaged organs and tissues. All studies included have reported favorable results in enhancing the migration of stem cells [27,29,48].

Finally, extensive research focused on the structural and biological optimization of scaffolds, evaluating cytotoxicity, proliferation, migration, odontogenic, and neurogenic differentiation to better replicate the dental pulpal microenvironment [32,39,40,44,45,49,59,62,65,68,70,73]. Furthermore, several studies demonstrated that biologically incorporating substances like growth factor into scaffold matrices significantly increased their regenerative performance [22,23,24,35,39,40,50,53,55,56,69,71]. Platelet-based scaffolds, obtained through autologous blood centrifugation, were also widely investigated to establish the ideal formulation for CF-RET success [19,38,47,52].

The data extraction of the included in vitro studies is reported in Table 2.

### 3.2. In Vivo Studies

Five papers were included in this narrative review, all investigating clinically applicable scaffold strategies for pulp regenerative procedures in traumatized necrotic immature teeth. Most studies focused on platelet-based scaffolds like i-PRF (injectable platelet-rich fibrin) and PRP (platelet-rich plasma) obtained through various centrifugation protocols, with the aim of identifying a material suitable for root canal application [75].

Overall, the available clinical evidence has consistently demonstrated a positive effect of platelet concentrates on regenerative outcomes.

Root development (assessed by measuring root length and canal diameter) [76,77,78], along with clinical presentation [77,78], showed significant results. No significant difference was found between groups except for the apical canal diameter (i-PRF showed significantly greater decrease in apical canal diameter than PRP) [75].

Beyond scaffold form, adjunctive biological factors were explored in a limited number of studies. Only one in vivo investigation quantified endogenous TGF-β, an essential growth factor released from the teeth following different irrigation protocols [9], highlighting its potential clinical relevance due to ease of application compared to other factors. Additionally, concentrated growth factor (CGF) supplementation was shown to provide clinically meaningful improvements when compared with non-supplemented scaffolds [78].

The data extraction of the included in vivo studies is reported in Table 3.

### 3.3. Quality Assessment Score

All the 64 articles included met the criteria of the quality assessment, resulting in reliability with a low risk of bias (Supplementary Table S1). The highest score was 36/36 [9,75], and the lowest was 18/36 [35]. The variability in methodological quality observed among the included studies, as highlighted by the QuADS assessment, underscores the need for a cautious interpretation of the findings, particularly given the heterogeneity of experimental models, interventions, and outcome measures. Consequently, the methodological appraisal should be considered supportive and interpretative, rather than definitive.

## 4. Discussion

The previous review by Eramo et al. [14] established that several cytokines, alone or in combination, have the potential to induce key biological effects (migration, proliferation, and differentiation) in dental stem cells and that various cell-homing molecules had successfully promoted pulp-like connective tissue formation in in vivo experiments [79,80]. However, it also concluded that dental pulp regeneration via cell homing still requires further investigation.

In line with topics addressed in previous studies, stem cell factors (SCFs) continue to be a major focus of investigation. They are considered potent chemokines involved in progenitor cell recruitment, as they have been observed in abundance at repair sites and are widely used in tissue engineering applications [54]. Consequently, most of the studies included aimed to identify molecules capable of mimicking the action of growth factors in order to enhance the ability of stem cells to reconstitute pulp tissue during CF-RET. Concentrated growth factor (CGF), the most recent generation of platelet concentrate products, is the most extensively investigated among the studies included in this structured narrative review. CGF contains key growth factors, including PDGF-BB, IGF-1, TGF-β1, bFGF and VEGF, which are released from platelets or autologous leukocytes, and is characterized by a fibrin network similar to that found in native tissues. These properties confer significant clinical potential [30], including the ability to promote pulp revascularization and increase root wall thickness [81,82].

The use of endogenous growth factors has also been shown to simplify clinical application, as they can be released from dental and periodontal tissues. In this context, dentin-derived matrix proteins have been reported to induce cell migration, regulate cell growth, and promote the differentiation of dental pulp cells [83]. However, the use of exogenous growth factors to stimulate healing remains associated with several technical and regulatory limitations, including high costs and potential safety concerns.

Among dentin-derived growth factors, TGF-β has been widely investigated due to its ability to modulate cell proliferation, differentiation, adhesion and migration. It can be applied at specific concentrations, which have yet to be fully defined, both exogenously and endogenously, and may be released from dentine walls by commonly used root canal irrigants, such as EDTA [9].

Although approaches involving platelet-derived concentrates, including CGF, are frequently investigated, the number of available studies does not necessarily correspond to the strength of evidence or clinical superiority. Most supporting data derive from in vitro experiments or small clinical series, which limits the robustness and generalizability of the reported findings.

To appropriately interpret these outcomes, it is essential to distinguish between true pulp–dentin regeneration and reparative or revascularization processes. A critical distinction in regenerative endodontics is that between true pulp–dentin complex regeneration and tissue repair or revascularization. True regeneration implies the re-establishment of an organized pulp tissue containing functional vasculature, neural elements, and a polarized odontoblast layer capable of physiological dentin deposition. In contrast, revascularization and repair typically result in the ingrowth of connective tissue, cementum-like or bone-like structures and limited or absent odontoblastic organization. When the in vivo human studies included in the present review are evaluated against this histological benchmark, it becomes evident that most outcomes are inferred from clinical and radiographic parameters, such as root lengthening, canal wall thickening, and periapical healing, rather than from direct histological evidence. While these findings indicate biological activity and functional improvement, they do not conclusively demonstrate the regeneration of a native pulp–dentin complex.

Critical insight into the nature of tissues formed after CF-RET is provided by seminal animal studies employing histological evaluation. In a well-characterized canine model, Palma et al. [84] demonstrated that regenerative endodontic procedures based on cell homing predominantly resulted in the formation of highly vascularized connective tissue associated with intracanal cementum-like deposits and, in some cases, bone-like tissue within the root canal space, rather than a structured pulp–dentin complex. Only limited and localized areas showed dentin formation with odontoblast-like cells, indicating that true pulp regeneration occurred inconsistently and was not the prevailing outcome. These histological findings are particularly relevant when interpreting clinical and radiographic success parameters reported in human studies, such as root lengthening, canal wall thickening, and apical closure. While these outcomes reflect biological activity and functional improvement, animal evidence suggests that they often arise from reparative processes involving cementogenesis or osteogenesis, rather than restoration of native pulp tissue architecture. Taken together, translational evidence from animal models indicates that CF-RET currently achieves reliable resolution of infection and promotion of tissue ingrowth but does not consistently fulfill the histological criteria of true pulp–dentin complex regeneration. This distinction is essential for avoiding overinterpretation of clinical success and for guiding future research toward strategies that promote organized pulp tissue formation, including innervation and odontoblast layer re-establishment.

It is also important to consider studies that have proposed the use of suitable scaffolds designed to provide stem cells with a regenerative microenvironment capable of mimicking the complex structure of dental pulp, thereby supporting stem cell development and proliferation. The use of materials such as decellularized human pulp [70,73], or dentine [62,65] has proven to be a successful strategy. These materials retain some of the natural properties of dental tissue by releasing bioactive substances and factors that promote regeneration, contributing to the creation of biomimetic environments that exploit the molecules responsible for the tooth’s innate self-repair capabilities and biological functions, including proliferation, migration, and odontogenic and neurogenic differentiation [85].

In addition, a careful evaluation of the scaffold microstructure and rheological properties is essential, as the physical and structural characteristics, such as substrate stiffness and geometry, directly influence cell behavior and their capacity to differentiate into multiple cell lines. In this context, photolithography has been used to fabricate a micropatterned substrate that encourages cells to adopt morphologies resembling odontoblasts, as their elongated shape, the presence of cellular processes, and palisade-like alignment appear to be fundamental to tooth physiology and the mineralizing phenotype [25,28]. Notably, Bordini et al. developed a physiologically relevant model using organoids to simulate dental pulp conditions without the need for animal models [22,23].

The review by Eramo et al. reported that odontoblastic and neural tissue regeneration has been achieved in only a limited number of studies, whereas current in vitro investigations more consistently focus on mineralization and the presence of odontoblasts through established assays. However, the standardization of methodologies aimed at evaluating nerve fiber formation and neurogenic regeneration remains a significant challenge. This suggests that, despite substantial progress in characterizing the dentin-forming phenotype, the complex requirements necessary to achieve functional pulp innervation are still incompletely understood and require more consistent and standardized experimental protocols in the scientific literature.

The findings of the present review should also be interpreted in the context of the systematic review and meta-analysis by Meschi et al. [86], which evaluated the clinical effectiveness of revitalization procedures in necrotic mature and immature permanent teeth. That analysis, based on a limited number of randomized and non-randomized clinical studies, reported no statistically significant differences in survival or success between revitalization and conventional endodontic treatments and rated the certainty of evidence as low to very low. These conclusions align with our observation that, despite encouraging biological and early clinical signals, the current human evidence does not support definitive clinical recommendations for regenerative endodontic therapies beyond carefully selected cases. Importantly, while the review by Meschi et al. focused on clinical outcomes of revitalization as a treatment modality, the present review addresses a complementary aspect by synthesizing recent in vitro and in vivo human studies specifically investigating cell-free regenerative endodontic strategies. Our findings, therefore, primarily inform biological feasibility, mechanistic understanding, and translational potential, rather than clinical effectiveness, which remains insufficiently supported by robust long-term evidence.

With regard to in vivo studies, compared with previous research, the present review identified clinical investigations that primarily focused on immature teeth, reporting improved outcomes in the main parameters evaluated. Previously, studies have suggested that the success of pulp regeneration depends on the clinical and biological conditions of the tooth, with cell-homing strategies not being equally effective in all scenarios, such as cases of pulp necrosis. Consequently, root canal treatment remains the standard of care for mature teeth with necrotic pulp and closed apices [14]. Consistent results for these specific clinical conditions have not yet emerged from the in vivo studies analyzed in this review.

More recently, in vivo investigations have begun to focus on clinical situations in which this type of therapy is both feasible and more likely to be successful, taking into account the initial condition of the tooth. Teeth from young patients that are not yet necrotic and present a larger exposed dentin surface release greater amounts of TGF-β, which can be exploited for regenerative purposes [9] and may be suitable for CF-RET treatment. To date, in vivo human studies on CF-RET have predominantly focused on immature teeth with incomplete root development; however, this trend reflects the current direction of clinical research, rather than definitive evidence, limiting the applicability of CF-RET to this patient group.

Although the available evidence indicates promising biological potential, its translational strength remains limited and is not yet applicable to routine clinical practice. Further studies are, therefore, required to investigate CF-RET in both immature and mature teeth and to clearly distinguish biological feasibility from clinical predictability. At present, evidence in immature teeth supports biological plausibility, rather than reliable and reproducible clinical outcomes, highlighting the need for dedicated clinical studies in adult patients with fully developed roots to expand the therapeutic indications of CF-RET.

Despite the significant progress made since the publication of Eramo et al., particularly in the development of biomimetic scaffolds and in the understanding of molecular mechanisms, a critical question remains unresolved. Although the reviewed studies collectively support the biological feasibility of CF-RET, the heterogeneity of study designs and the predominance of in vitro evidence limit the strength of clinical inferences. The current findings should, therefore, be interpreted as indicative of regenerative potential, rather than as proof of predictable clinical effectiveness. While both in vitro and in vivo results are encouraging, standardized protocols for CF-RET procedures in humans are still lacking. Such protocols are essential to ensure consistent reproducibility across different clinical settings and operators, which is a prerequisite for the safe and effective application of CF-RET.

Moreover, substantial variability in experimental conditions, scaffold composition, growth factor concentrations, and outcome assessment across studies hampers direct comparison and weakens the overall consistency of the evidence. Consequently, although biological feasibility is well supported, the clinical translatability of CF-RET remains confined to selected scenarios (primarily immature teeth) and should be regarded as investigational, rather than predictive of routine clinical success.

### Study Limitations

Although this review incorporated structured methodological elements such as protocol registration in PROSPERO, a predefined search strategy, and transparent reporting of study selection, it was ultimately conducted as a structured narrative review. This approach was justified by the substantial heterogeneity of the included studies, which precluded meaningful quantitative synthesis or meta-analysis. PRISMA 2020 guidelines were used to guide reporting of the search and selection processes, while PROSPERO registration was undertaken to ensure methodological transparency and avoid duplication. Consequently, the evidence was synthesized using a qualitative, narrative approach.

A major limitation of this review is the pronounced heterogeneity in methodologies and reported outcomes across the included studies. This variability limited the feasibility of statistical aggregation and meta-analyses. In addition, only a small number of in vivo human studies were available, all of which focused on immature teeth, thereby restricting the generalizability of the findings.

The methodological quality of the included studies, assessed using the QuADS tool, also showed considerable variability. Differences in study design, reporting completeness, and experimental approaches indicate that the findings should be interpreted with caution, particularly when extrapolating in vitro results to clinical contexts.

Another limitation is the absence of studies systematically investigating the combined use of multiple key components of CF-RET, such as scaffolds, growth factors, and endogenous stem cells’ recruitment, either in vitro or in vivo. Consequently, the relative contribution of each component and the potential synergistic effects of specific combinations remain poorly understood. In addition, few studies incorporated microbiological analyses to evaluate scaffold-related bacterial growth alongside stem cell proliferation [24], and only a limited number examined cell morphology, spatial organization, or the use of scaffolds derived from natural and appropriately processed human tissues.

Finally, ethical constraints severely limit the feasibility of histological validation of regenerated tissues in human teeth, representing a major challenge in the interpretation of clinical regenerative endodontic studies. As a result, current evidence supporting CF-RET largely reflects reparative or revascularization processes, rather than confirmed true pulp–dentin complex regeneration. This underscores the need for caution when interpreting regenerative claims.

Future translational research should aim to bridge this gap by integrating advanced imaging, molecular markers, and validated surrogate endpoints that more accurately reflect pulp tissue organization and functionality, to determine whether emerging CF-RET strategies can consistently achieve true regeneration, rather than tissue repair alone.

An additional constraint of the present structured narrative review is the inclusion of studies combining stem cells with specific molecules or microcapsules. This is relevant because, unlike CB-RET, CF-RET relies on endogenous cells migrating from periapical tissues, rather than the direct placement of cells into the root canal in combination with scaffolds and growth factors [71].

## 5. Conclusions

In recent years, interest in developing a valid alternative to the conventional treatment of necrotic teeth has increased substantially. In vivo studies evaluating materials previously investigated in vitro have reported encouraging clinical and radiographic outcomes, suggestive of pulp–dentin tissue formation. However, further studies are clearly required to establish standardized methodologies and clinical guidelines for regenerative endodontic therapy, as well as to assess long-term outcomes. CF-RET represents a promising regenerative strategy, particularly in immature teeth with incomplete root development. Nevertheless, its broader clinical application remains investigational and is not yet supported by robust, long-term clinical evidence.

The heterogeneity observed in reported outcomes and clinical indications is further reflected in recent multinational surveys demonstrating substantial variability in RET adoption. These findings highlight the need for standardized clinical protocols, targeted professional training, and well-designed translational and clinical studies to support the safe and predictable implementation of CF-RET in clinical practice.

## Figures and Tables

**Table 1:** PICOS format.

**Population**	Stem cells (primary cultures and human cell lines); patients with mature or immature permanent teeth requiring regenerative pulp treatment due to trauma or endodontic injury
**Intervention**	Pulp regeneration via cell homing (CF-RET)
**Comparison**	No pulp regeneration via cell homing
**Outcome**	In vitro studies: cell viability, proliferation, attachment, cytoskeletal cell morphology, metabolic cell activity, adhesion, mineralized matrix deposition, chemotactic potential, cytotoxicity, apoptosis, cell cycle analysis and colony forming unit (CFU). Cell differentiation in scaffolds and the antimicrobial effect of the scaffolds. Odontogenic differentiation. Migration activity. Migratory speed rate evaluation. Gene expression of specific genes related to osteogenesis and pulp regeneration, such as MMP9, OCN, OPN and TGF-β, OSX, DLX5, MSX2 and BMP2. Activation of WNT/β-catenin and JNK signaling pathways. TGF-β1 expression in various concentrations of A-PRF in the differentiation process of hDPSCs. Analysis of TAZ and miR-584. Luciferase activity. Angiogenesis on HUVECs and SCF + HUVECs groups. Expression of odontogenic markers such as ALP, OCN, ON, OP, RUNX-2, DSPP, DMP-1, ANG, CXCR4, VEGF and PDGFRA, BSP, ITGA5, ITGAV, COL1A1, COL3A1, β–III–Tubulin, Factor VIII, KDM4D, RPS5, GAPDH, HA, PRMT6, VEGF-A expression of hDPSCs. Accumulation of phospho-Smad 2/3 in cells. Cell polarization. Endocytosis by SCAP and HUVECs. Differential expression of miRNAs between DPSCs and SCAP. Protein expression PAI-1, uPA, suPAR, uPAR. Expression levels of AKT, miR-9 and KLF5, or determine the effect of miR-9. Chemotaxis of SCAPs. In vivo studies: pulp sensitivity, root length and canal diameters, periradicular lesions, spontaneous pain, pain on palpation, sinus tract, swelling and crown discoloration, functional retention, root width, root resorption area and all root area minus pulp space.
**Study Design**	In vitro and in vivo human studies

**Table 2:** In vitro studies selected.

Author/Authors and Year	Cell Populations	CF-RET Intervention	Biological Effects	Assay Used and Conditions	Principal Findings
**Abdelgawad et al. (2021)** [17]	Human dental pulp stem cells (HDPSCs)	Photobiomodulation with diode laser (PBM) and vitamin D, groups: control, vitamin D (10^−7^ Mol); irradiation at 1 J/cm^2^ of 810 nm diode laser; irradiation at 1 J/cm^2^ and culture with vitamin D; irradiation at 2 J/cm^2^, irradiation at 2 J/cm^2^ and culture with vitamin D	Viability, proliferation, odontogenic differentiation, osteogenesis, and pulp regeneration gene expression (MMP9, OCN and TGF-β)	MTT assay (viability and proliferation), alkaline phosphatase production (ALP) activity and ARS staining (odontogenic differentiation), RT-qPCR (gene expression)	PBM at 1 and 2 J/cm^2^ combined with vitamin D enhanced the HDPSCs proliferation and odontogenic differentiation (*p* < 0.0001)
**Al-Ateeq et al. (2024)** [18]	HDPSCs	Polydatin (PD) 0.01 µM, 0.1 µM, and 1 µM concentrations	Viability, ALP, odontogenic differentiation, mineralization, odontogenic markers expression (ALP, OCN, osteonectin (ON), osteopontin (OP), RUNX-2, DSPP, and DMP-1)	Alamar blue^TM^ assay (Thermo Fisher Scientific, Waltham, MA, USA) for 1, 3, and 7 days (viability). ALP activity for 7 and 14 days, and ARS staining for 14 and 21 days, RT-qPCR	PD significantly enhanced viability of HDPSCs at 0.01 µM (*p* = 0.043) and 0.1 µM on day 7 (*p* = 0.010). 0.01 µM (*p* = 0.003), 0.1 µM (*p* = 0.010), and 1 µM (*p* = 0.005) increased expression of markers associated with odontogenic differentiation. ALP activity of HDPSCs was significantly increased with 0.01, 0.1, and 1 µM of PD. There was a notable increase in mineralized tissue deposits in the presence of PD.
**Bagio et al. (2021)** [19]	HDPSCs from ten third molars extracted from nine healthy donors	10 mL of blood was collected and processed to obtain Advanced Platelet-Rich Fibrin (A-PRF) gel at 1%, 5%, 10% A-PRF for the conditioned media (CM) of experimental groups. Control group: hDPSCs + DMEM (Dulbecco’s Modified Eagle Medium)	VEGF-A expression of hDPSCs observed for 5, 12, 24 h	VEGF-A ELISA at 405 nm for 5, 12, and 24 h after treatment	5% A-PRF CM was superior in increasing VEGF-A expression of hDPSCs at 5, 12 and hours of observation (*p* < 0.05)
**Bagio et al. (2024)** [20]	Primary hDPSCs	Media supplemented with 0.1 mg/mL and 1 mg/mL of calcium hydroxide (Ca(OH)_2_) and triple antibiotic paste (TAP: mixture of ciprofloxacin, metronidazole, and minocycline in a 1:1:1 ratio), and a combination of Ca(OH)_2_ and TAP at equivalent concentrations, with DMEM serving as the control group	Cell viability	MTT assay	Notable variation in hDPSCs viability observed among all groups, with the lowest viability recorded in the combination of Ca(OH)_2_ + TAP at 1 mg/mL (*p* < 0.05). TAP alone, at concentrations of 0.1 mg/mL and 1 mg/mL, displayed better cell viability compared to Ca(OH)_2_. The combination of Ca(OH)_2_ and TAP at a concentration of 0.1 mg/mL improved hDPSCs viability compared to higher concentrations, indicating a potentially beneficial synergistic effect at lower doses.
**Bagio et al. (2023)** [21]	HDPSCs from nine third-molar teeth extracted from healthy donors	HDPSCs + DMEM; hDPSCs + DMEM supplemented with 10% fetal bovine serum (FBS); hDPSCs + 10% platelet-rich plasma-thrombin (PRP-T); hDPSCs + 0.5% thrombin-activated platelet-derived exosome (TaPDE), hDPSCs + 1% T-aPDE; and hDPSCs + 5% TaPDE	Cell viability, migration activity, VEGF-A expression	MTT essay (viability), scratch essay (migration), and ELISA (VEGF-A expression)	The 5% T-aPDE group (*p* < 0.05) demonstrated the highest viability absorbance value of hDPSCs after 24, 48, 72 h of observation (*p* < 0.05), and the closest distance result of migratory activation hDPSCs (*p* < 0.05), and the highest VEGF-A expression of hDSPCs was noted at 72 h observation (*p* < 0.05).
**Bordini et al. (2022)** [22]	3D culture of dental pulp cells (DPCs) in a pulp-in-a-chip platform under simulated pulp pressure organoid	DPCs directly cultivated on chitosan (CH), CH-Ca- βGP Chitosan-based scaffold combined with calcium hydroxide and β-glycerophosphate (βGP) (CH-Ca-βGP), CH-Ca, and CH-βGP	Attachment, cytoskeletal cell morphology, metabolic cell activity, cell viability, adhesion, alkaline phosphatase, mineralized matrix deposition, chemotactic potential	Live/dead assay (Thermo Fisher Scientific, Waltham, MA, USA) up to 14 days (attachment). F-actin assay (cytoskeletal call morphology), and Alamar Blue (Thermo Fisher Scientific, Waltham, MA, USA) (metabolic cell activity up to 21 days and cell viability at 1, 3, 7, and 14 days). ALP and Alizarin Red (mineralized matrix deposition) up to 21 days. o-cresolftalein method at 14 days (calcium deposition), Transwells polyethylene membrane (chemotactic potential), DSP immunofluorescence, Fluorescence staining with DAPI reagent (cell adhesion)	The CH-Ca-βGP formulation significantly increased cell viability during early cultivation periods. It was the only formulation that enhanced mineralized matrix deposition and induced chemotaxis compared to plain chitosan scaffolds.
**Bordini et al. (2024)** [23]	Human DPC in a microchip, under simulated dental pulp pressure organoid	CH and chitosan-calcium aluminate (CHAlCa) scaffold	Cell viability, migration, proliferation, presence of dentin sialoprotein, in situ mineralized matrix deposition, and odontoblastic differentiation markers: ALP, DMP1, DSPP and collagen type I alpha 1	LIVE/DEAD^®^ viability/cytotoxicity assay (Thermo Fisher Scientific, Waltham, MA, USA), and Alamar Blue (Thermo Fisher Scientific, Waltham, MA, USA) reagent (proliferation) after 1, 3, 7 and 14 days, immunofluorescent staining for F-actin filaments and DAPI for nuclei on days 7 and 14 (migration and presence of dentin sialoprotein), Alizarin Red staining (in situ mineralized matrix deposition) at 14 days, and qPCR.	Incorporation of calcium aluminate into the chitosan scaffold significantly (*p* < 0.05) improved its bioactive properties, leading to better recruitment and the differentiation of human dental pulp cells. The study indicated upregulation of odontoblastic markers and enhanced mineralized matrix deposition in cells interacting with the CHAlCa scaffold.
**Caballero-Flores et al. (2021)** [24]	Human stem cells from apical papilla (hSCAPs) of immature impacted caries–free third molars with open apices	Scaffolds based on CH; CH associated with gelatin and genipin (ChGG); Chitosan associated with gelatin, microparticulate dentin and genipin (ChGDG)	Cell morphology and adhesion to the scaffold, cytotoxicity and cell proliferation, cell differentiation in scaffolds, and the antimicrobial effect of the scaffolds	SEM at 2 and 8 d (cell morphology and adhesion), MTT assay at 24 and 48 h (cytotoxicity and cell proliferation), alizarin red assay at 21 d (cell differentiation), and bacterial culture on m-Enterococcus agar at 6, 24 and 48 h (antimicrobial effect).	ChCDG had more protein release, adhesion, proliferation and differentiation of hSCAPs. The addition of microparticulate dentin significantly enhances the biological properties of the scaffold and promotes antibacterial activity against residual bacteria in infected root canals (Enterococcus faecalis) than CH and ChGG.
**Chang et al. (2020)** [25]	hDPSCs	Nanofibrous tubular three-dimensional (3D) platform	Change in the cellular process length over time and change in the ratio of single DPSC	SEM	Nanofibrous, tubular structure of the 3D matrix is crucial in initiating DPSC polarization. Compared to compared to the group of 12 h, change in the cellular process length is significant at 24 h (*p* < 0.05) and 48, 72, 96 h (*p* < 0.01) and change in the ratio of single DPSC with a process is significant at 48, 72 and 96 h (*p* < 0.05).
**Chang et al. (2020)** [26]	SCAPs of immature permanent teeth extracted for orthodontic reasons	Various concentrations of TGF-b1 (0.5, 1, 5, 10, and 25 ng/mL)	Cell viability, protein expression of plasminogen activator inhibitor-1 (PAI-1), urokinase-type plasminogen activator (uPA), soluble urokinase-type plasminogen activator receptor (suPAR), uPA receptor (uPAR), and effect of SB431542 (ALK5/Smad2/3 inhibitor) on TGF-b1-induced changes of uPAR and PAI-1 protein expression	MTT assay (viability) at 2 h, and Western blotting and ELISA at 5 days (protein expression)	TGF-b1 significantly (*p* < 0.05) influences the plasminogen activation (PA) system in SCAPs: stimulates the secretion of PAI-1 at 0.5, 1, 5, 10, and 25 ng/mL, and uPAR at 0.5, 1, 5, 10, and 25 ng/mL, and inhibiting uPA secretion at 1, 5, 10 ng/mL. The study noted that the treatments with TGF-b1 did not markedly alter the cell viability of SCAPs, indicating that TGF-b1 can exert its regulatory effects without adversely affecting the survival of these stem cells.
**Chen et al. (2022)** [27]	SCAPs	Exosomes were isolated from the dental pulp tissue (DPT-exos) and stem DPC-exos of swine	Endocytosis by SCAP and HUVECs, cell migration, promotion proliferation, differentiation and expression of odontogenic proteins	CKK-8 (Sigma-Aldrich, St. Louis, MO, USA), Transwell, angiogenesis, and odontogenic induction assays	DPT-exos exomes have been shown to promote the migration, proliferation and differentiation of SCAPs.
**Ha et al. (2020)** [28]	SCAPs derived from human third molars	Gelatin methacryloyl (GelMA) hydrogels of increasing concentrations (5, 10 and 15%) and cultured for up to 7 days	Cell spreading, alignment, viability, morphology, proliferation, and differentiation	F-actin/DAPI immunostaining. Live/Dead stain (Thermo Fisher Scientific, Waltham, MA, USA), ActinGreen/NucBlue assay kit (morphology), ALP activity (differentiation)	Both 60 and 120 micropatterned hydrogels guided the self-alignment of SCAPs with no significant difference between them (*p* < 0.001), promoted significant (*p* < 0.01) higher odontogenic differentiation than non-patterned controls.
**Hong et al. (2018)** [29]	hSCAP normal impacted third mandibular molars without apical closure collected from 3 healthy patients	Three concentrations of 1 Concentrated growth factor (CGF) or 1 platelet-rich fibrin (PRF) (1 CGF or PRF membrane dissolved in 10 mL DMEM), 1/2 CGF or 1/2 PRF, or 1/4 CGF or 1/4 PRF were used)	Cell proliferation, migration, mineralization, and differentiation by the expression level of ALP, DMP1, DSPP, BSP	Cell Counting Kit-8 (Sigma-Aldrich, St. Louis, MO, USA) at 7 days (proliferation), transwell assays for 12 and 24 h (migration), Alizarin Red S staining at 7 and 14 d (mineralization), and qPCR (differentiation)	Both CGF and PRF can promote the proliferation at every time point (*p* < 0.05), migration for 12 h (*p* < 0.05), and differentiation of SCAPs at 14 days. No significant difference between the CGF and PRF groups. The mineralized areas in the CGF and PRF groups were more significant than those in the control group after incubation for 7 days (downregulated) and 14 days (upregulated) (*p* < 0.05). The related gene expression levels in the PRF group were higher than those in the CGF group.
**Hong et al. (2019)** [30]	hSCAP from unerupted third mandibular molars without apical closure	CGF dissolved in 10 mL DMEM (supplemented with 10% FBS) were defined as 1 X CCM; thereafter, three different concentrations of CCM (1/4 X CCM, 1/2 X CCM and 1 X CCM) were designed	Cell proliferation, migration, mineralized nodule formation, and gene expression of ALP, DSPP and DMP-1	Cell Counting Kit-8 (Sigma-Aldrich, St. Louis, MO, USA) at 2 days (proliferation), transwell assays for 24 h (migration), Alizarin Red S staining at 7 and 14 days (mineralization), and qPCR (gene expression)	Compared to the control group CGF significant (*p* < 0.05) improves growth rate and migratory cell numbers, mineralization areas, and expression levels of ALP, DSPP and DMP-1.
**Jiang et al. (2020)** [31]	hDPSCs	Cells were treated using different concentrations of liposomal TGF-β1, with a lipid concentration of 100 μg/mL and final encapsulated TGF-β1 concentrations of 0 ng/mL, 1 ng/mL, 5 ng/mL and 10 ng/mL, respectively. Free TGF-β1 (5 ng/mL) and PBS treatment were used as controls.	Cell viability, odontogenic differentiation, gene expression of RUNX-2, DMP-1, and DSPP	MTT assay after 1, 3 and 7 d (viability), Alizarin Red S staining at 21 d, RT-qPCR at 7 d, and ELISA at 7 d (odontogenic differentiation and gene expression)	Liposomes (100 μg/mL) were not cytotoxic to the cells and allow a controlled release of TGF-β1. For the free TGF-β1 group, the cell viability was 118% at 7 days, which was higher than the other groups (*p* < 0.05), reflecting the TGF-β1 induced proliferation of hDPSCs. Liposomal TGF-β1 upregulated the expression of “osteodentine” markers, RUNX-2, DMP-1 and DSPP, in hDPSCs after 7 days of treatment for all groups (*p* < 0.05). Representative pictures of the accumulation of mineralized nodules were taken.
**Jin et al.** **(2023)** [32]	hDPSCs from tooth extractions from patients ranging from ages 18–25 years	hDPSCs were cultured on various types of collagen scaffolds, including bare collagen scaffold (BCS), mineralized collagen scaffold (MCS), or 1.2 mg/mL hyaluronic acid (HA) modification of MCS (HA-MCS)	Cell viability, morphology, and odontogenic differentiation	Live/dead assay kit (Thermo Fisher Scientific, Waltham, MA, USA) at 48 h (viability), SEM with immunofluorescence staining at 2 d (morphology), ALP activity and Alizarin Red S staining for 7 and 14 days (differentiation)	The scaffolds, particularly the HA-MCS, significantly enhanced the proliferation and differentiation of hDPSCs. This was evidenced by a significant (*p* < 0.01, and *p* < 0.001) increase in ALP activity at different time points, particularly on day 14 (*p* < 0.05 between HA-MCS and MCS), confirmed by Alizarin Red S staining, indicating that the mechanical microenvironment provided by the HA-MCS is conducive to odontogenic differentiation.
**Karkehabadi et al. (2023)** [33]	hSCAP from undeveloped root of a third molar of a human tooth	10 μg/mL of melatonin (N-acetyl-5-methoxytryptamine) to the experimental group cells while they were cultured in an osteogenic medium. The control group did not receive any treatment.	Cell viability, odontogenic/osteogenic differentiation, and expression of osteogenic genes, including ALP, DSPP, DMP1, BSP	MTT assay on days 1, 2, and 3 (viability). ALP activity, alizarin red staining (ARS) at 7 and 21 days (differentiation), and qRT-PCR on days 1, 2, and 7 (gene expression).	After 1, 2, and 3 days, no significant difference was observed between the control group and the melatonin treatment group in terms of viability of hSCAPs. (from 1 up to 10 μg/mL) (*p* > 0.05). The assessment of ARS and ALP activity showed that melatonin treatment enhanced osteogenic differentiation of hSCAPs (*p* < 0.001). Melatonin treatment resulted in a significant (*p* < 0.001) increase in the expression of osteogenic and odontogenic markers.
**Ke et al. (2019)** [34]	hDPSCs and hSCAPs from human dental pulp tissues. and dental apical tissues	Transinfecting cells with miR-224 mimic and his negative control (NC), miR-224 inhibitor and his inhibitor negative control	Cell migration and proliferation, osteogenic differentiation and differential expression of miRNAs between DPSCs and SCAP	CCK-8 at 6 d (proliferation), transwell assay after 24 h (migration), Alizarin Red Staining and ALP detection kit after 21 days (osteogenic differentiation), quantitative Real-Time PCR (expression of miRNAs)	SCAPs showed a greater capacity for proliferation and osteogenic differentiation compared to DPSCs. Downregulation of miR-224-5p increased the migration and proliferation of DPSCs. -Proliferation of SCAPs: CGF vs. Control (*p* < 0.05), PRF vs. Control (*p* < 0.05), CGF vs. PRF (no significant difference). - Migration of SCAPs after 12 Hours: PRF vs. Control (*p* < 0.05), CGF vs. Control (no significant difference). - Migration of SCAPs after 24 Hours: CGF vs. Control (*p* < 0.05), PRF vs. Control (*p* < 0.05), CGF vs. PRF (no significant difference). - Mineralization: CGF vs. Control (*p* < 0.05), PRF vs. Control (*p* < 0.05), CGF vs. PRF (No direct comparison *p*-value provided). - Expression of Odontogenic Markers (ALP, BSP, DMP-1, DSP): PRF vs. CGF (*p* < 0.05), CGF vs. Control (*p* < 0.05, inferred from context for CGF vs. control).
**Kim et al. (2022)** [35]	DPCs	Hyaluronic acid (HA)-collagen hybrid hydrogel with controlled release of fibroblast growth factor (FGF-2: 100, 200, 500, and 1000 ng/mL) and platelet-derived growth factor (PDGF-BB: 10, 50, 100, 200, and 500 ng/mL) were experimented on days 1, 3, 5, and 7	Cell viability and proliferation. The release kinetics of growth factors (30 ng/hydrogel) from the HA-collagen hydrogel.	Live/dead assay (Thermo Fisher Scientific, Waltham, MA, USA). Cell counting kit (CCK)-8 kit (Sigma-Aldrich, St. Louis, MO, USA) at 1, 3, 5, and 7 days (proliferation). ELISA (release kinetics)	Considering FGF-2 concentration, significantly (*p* < 0.05) increased pulp cell proliferation was observed on days 1, 3, 5, and 7 in the 100 ng/mL group and on days 3, 5, and 7 in the 200 ng/mL group. In the case of PDGF-BB concentration, significantly (*p* < 0.05) increased pulp cell proliferation was observed at all four time points in the 100 ng/mL group and on days 3, 5, and 7 in the 50, 200, and 500 ng/mL groups. No significant cytotoxicity was found.
**Kornsuthisopon et al. (2022)** [36]	Immortalized human dental pulp stem cell (ihDPSC)	Various concentrations of betaine (BET) were used, specifically 1, 5, 10, 50, 100, 250, and 500 μM. The growth medium for the cells included 100 units/mL penicillin, 100 μg/mL streptomycin, and 250 ng/mL amphotericin B.	Cell Proliferation, ALP and mineralization, adipogenic differentiation. Gene expression of osteogenic-related genes RUNX2, OSX, DLX5, MSX2, ALP, and BMP2.	MTT assay on 1, 3, and 7 days (proliferation), oil red O for 16 days (adipogenic differentiation), ALP activity and Alizarin Red S (ARS) staining at 14 days (mineralization). RT-qPCR (gene expression)	A significant decrease in cell proliferation was found in 100, 250, and 500 mM BET treatment on day 3 (*p* = 0.0226, *p* = 0.0213, *p* = 0.0005, respectively) Treatment with 50 mM BET significantly promoted a significant increase in ALP and ARS staining detected after 7 and 14 d (*p* = 0.0286), and upregulation of osteogenic-related genes (*p* = 0.0022, *p* = 0.0022, *p* = 0.0152, *p* = 0.0022, *p* = 0.0022, *p* = 0.0022, respectively). The study highlighted that BET-induced osteogenic differentiation was mediated through intracellular calcium regulation.
**Lay et al. (2023)** [37]	hDPSCs	L-arginine at four different concentrations: 250, 300, 350, and 400 μmol/L	Cell viability	MTT assay at 24 h (viability)	Concentrations of 250, 300, and 350 μmol/L significantly (*p* = 0.007 between the control group and the treatment group) enhanced the viability compared to a control medium, with 400 μmol/L showing the best results. The concentration of 400 μmol/L had no significant difference (*p* > 0.05) with the control group.
**Le et al. (2024)** [38]	hSCAPs	The study did not explicitly mention the concentrations of advanced platelet-rich fibrin plus (A-PRF^+^) and i-PRF used in the experimental culture media. Positive control (PC) medium: DMEM/F12 supplemented with 10% fetal bovine serum and negative control (NC) medium: DMEM/F12.	Cell Proliferation, migration and gene expression of ALP, BSP, DSPP and DMP-1	Hemocytometer on 2, 4, 6, 8, and 10 days (proliferation), scratch-wound assay at 1, 2 and 3 days (migration), agarose gel electrophoresis (AGE) and RT-qPCR on 7 and 14 days (gene expression)	Both A-PRF^+^ and i-PRF were found to induce the proliferation, migration, and differentiation of SCAPs. However, in the i-PRF group, the cell number was significantly (*p* < 0.01) lower than that of the A-PRF+ group on days 8 and 10; the percentage of the scratched area on days 1 and 2 was significantly higher than in the A-PRF+ group (*p* < 0.05). The mRNA expression levels of biomarkers in the i-PRF group were similar to those in the A-PRF+ group.
**Leite et al. (2021)** [39]	hAPCs of 4 healthy third molars with incomplete root formation	Collagen (positive control); Collagen/gelatin (Col/Gel) 4:6; Col/Gel 6:4; Col/Gel 8:2 some of these were loaded with fibronectin (FN) (0, 5, or 10 microg/mL)	Cell viability, adhesion, spreading and migration and gene expression of ITGA5, ITGAV, COL1A1, and COL3A1	Alamar Blue (Thermo Fisher Scientific, Waltham, MA, USA) and Live Dead staining (Thermo Fisher Scientific, Waltham, MA, USA) (viability), microscopy to detect F-actin (adhesion and spreading), transwell assay and (migration), and RT-PCR (gene expression)	Cell viability in Col/Gel 8:2 and collagen (control) groups was similar at 1-, 3-, and 7-day time intervals (*p* < 0.05). The increase in gelatin ratio in the hydrogel formulations reduced the viability of hAPCs in comparison with the control (*p* < 0.05). Significant images of live/dead assay and detection of F-actin were taken. Incorporation of FN into the hydrogels increased the cell viability in all time intervals (*p* < 0.05). Collagen/gelatin hydrogel with 10 mg/mL of FN it has the highest bioactive, chemoattractant level on cells and favorable gene expression (*p* < 0.05).
**Leite et Al. (2022)** [40]	hAPCs of four third molars with incomplete root formation donated from different patients	Tubular scaffold (TB-SC) made from poly(caprolactone) (PCL) aligned nanofibers combined with a fibronectin (FN)-loaded collagen hydrogel. TB-SC without treatment, TB-SC coated with 10 μg/mL of FN (TB-SC + FN); TB-SC associated with collagen hydrogel (TB-SC + H); TB-SC associated with FN-loaded collagen hydrogel (TB-SC + HFN).	Cell proliferation, migration and gene expression of markers related to pulp regeneration (ITGA5, ITGAV, COL1A1 and COL1A3)	Alamar Blue (Thermo Fisher Scientific, Waltham, MA, USA) solution at 1, 7, and 14 days (proliferation), transwell assay and staining the cell cytoskeleton with an Actin Red 555 probe (migration) and RT-qPCR at 7 days (gene expression)	In all groups were noted an enhanced the migration, proliferation, and gene expression of markers associated with pulp regeneration of hAPCs compared to TB-SC group (*p* < 0.05). The hAPCs in the TB-SC + HFN group showed the highest values of cell proliferation and gene expression of COL1A1 and COL3A1 (*p* < 0.05), as well as superior cell migration results to groups TB-SC and TB-SC + H (*p* < 0.05).
**Li et al. (2018)** [41]	hDPSCs from extracted third molars	Transinfecting cells with siRNA targeted against Cdc42 (cell division control protein 42). Control: The scrambled siRNA was transfected as a negative control.	Cell migration, polarization, adhesion, and expression of related proteins	Scratch-wound assays at 36 h and transwell assays at 24 h (migration), staining of the various cellular organelles with immunofluorescence staining and visualization with a confocal microscope (polarization), and Western blot after 48 h (protein expression)	Silencing of Cdc42 inhibited the migration of hDPSCs and decrease in the proportion of polarized hDPSCs during migration (*p* < 0.01). Western blot analysis revealed that these effects depend on FAK, β-catenin and GSK3β proteins (*p* < 0.05).
**Liang et al. (2023)** [42]	Primary SCAPs	Manipulated expression of lysine demethylase 4D (KDM4D) in cells and the ribosomal protein encoded by RPS5	Mineralization, migration and chemotaxis of SCAPs. Expression of KDM4D, RPS5 and 3-phosphate Dehydrogenase (GAPDH).	ALP activity assay at 5 days and alizarin red staining at 2 w (mineralization), Western blotting (protein expression), scratch migration assays and transwell essay at 24 and 48 h (migration)	KDM4D was found to significantly (*p* < 0.01) promote the osteo/dentinogenic differentiation and migration of SCAPs at 24 (*p* < 0.05) and 48 h (*p* < 0.01). RPS5 enhanced osteo/dentinogenic differentiation (*p* < 0.01) and migration potential (*p* < 0.05) of SCAPs. KDM4D was shown to interact with the ribosomal protein RPS5.
**Liu et al. (2021)** [43]	hDPSCs isolated from extracted healthy permanent teeth	hDPSCs were cultured in the absence, or presence of different concentrations of Treated dentin matrix (TDM) (40%, 60%, 80%, 100%)	Odontogenic differentiation, cell migration, proliferation, and protein expression of DSPP, DMP-1, ALP, β–III–Tubulin, Factor VIII and RUNX2	Oil red O and alizarin red staining methods were performed on day 14 and 21, respectively (differentiation), EdU and CCK-8 assays (proliferation), immunofluorescence cell staining, transwell assays (migration), q RT-PCR. Western blot (protein expression).	All TDM leaching solution concentrations were significantly proven to promote odontogenic differentiation, proliferation and protein expression of hDPSCs.
**Liu et al. (2021)** [44]	hDPSCs from healthy premolars or third molars	Self-assembled peptide RAD/Dentonin hydrogel scaffold	Biocompatibility. Cell proliferation, odontogenic differentiation, expression levels of ALP, DSPP, OPN, and OCN, Runx-2	Immunofluorescent staining (cytoskeleton staining), CCK-8 at 1, 3, 5, and 7 days (proliferation), Live/Dead fluorescent staining (Thermo Fisher Scientific, Waltham, MA, USA) at 1 and 7 days (biocompatibility) and Calcein AM cell viability assay kit (proliferation). ALP activity assay at 7 and 14 days, RT-qPCR, and Alizarin Red S staining at 21 days (odontogenic differentiation).	The self-assembled peptide hydrogel scaffold demonstrated high biocompatibility and enhanced adhesion, proliferation (*p* < 0.01), and migration. The RAD/Dentonin functionalized hydrogel scaffold significantly promoted the expression of osteogenic and odontogenic genes. hDPSCs cultured on RAD/Dentonin scaffolds formed more and larger calcium nodules compared to those on RAD scaffolds and significant (*p* < 0.01) activation of ALP at 7 and 14 days.
**Loukelis et al. (2023)** [45]	DPSCs from healthy third molars	1. Kappa-carrageenan/chitosan/gelatin enriched with KCl (KCG-KCl) as a physical crosslinker; 2. kappa-carrageenan/ chitosan/gelatin (KCG); 3.chitosan/gelatin (CG) scaffolds as a control	Cell viability, ALP activity and gene expression of RunX2, ALP and DSPP. The concentration of secreted calcium secreted by the DPSC	CLSM live/dead fluorescent staining at 72 h, PrestoBlueTM cell viability assay (Thermo Fisher Scientific, Waltham, MA, USA) at days 3, 7 and 14 (viability). ALP activity essay and RT-PCR at 3, 7 and 14 days (gene expression). O-cresol phthalein complexone (CPC) method (calcium concentration) every three days of cell culture, up to day 28.	Cell viability data showed a significant increase from days 3 to 7 and up to day 14 for all scaffold compositions. Significantly increasing ALP activity has been observed over time in all three scaffold compositions. KCG-KCI and KCG exhibited great biocompatible and odontogenic differentiation capabilities. RT-PCR analysis revealed an earlier expression of the ALP marker for the two scaffolds, compared to the control, while KCG-KCl samples had the highest upregulation of the odontogenesis-specific DSPP marker at day 14. KCG-KCl scaffolds indicated significantly higher calcium production after 21 and 28 days. Compared with the control, the expression levels of ALP, Runx2, and OCN in the si-Cdc42 group were significantly reduced (*p* < 0.01). Alizarin Red S staining showed that mineralized nodules were significantly reduced in the si-Cdc42 group compared with the control group (*p* < 0.01). Compared with the control, the expression levels of p-PI3K, p-AKT and p-mTOR in the si-Cdc42 group were significantly decreased (*p* < 0.01). Compared with the control, the expression levels of p-PI3K, p-AKT, and p-mTOR in the si-Cdc42 + SC79 group were significantly increased (*p* < 0.01).
**Lv et al. (2018)** [46]	DPSCs from human third molars of patients	HDPCs were treated with 100 ng/mL recombinant human WNT7B (rhWNT7B)	Cell proliferation, migration. Activation of WNT/β-catenin and JNK signaling pathways. Odontogenic differentiation by protein expression, ALP activity, mineralization and gene expression of GAPDH, RUNX2, ALP, COL1.	Cell counting kit-8 (CCK-8 and 5-ethynyl-2′-deoxyuridine (EdU) assay), and immunofluorescence staining of Ki67, Annexin V staining (proliferation). Scratch assay at 24 h (migration). ALP activity at 7 d, alizarin red staining, qPCR, Western blot and dual luciferase assays at 48 h (odontogenic differentiation).	rhWNT7B treatment inhibited hDPCs and cell proliferation without affecting apoptosis, particularly at 100 ng/mL (*p* < 0.05), increased ALP activity and alizarin red staining, indicating increased osteogenic differentiation. WNT7B promoted the migration of HDPCs (*p* < 0.001). Finally, Runx2 (*p* < 0.01) and Col1 (0.0001) gene expression was elevated, suggesting activation of WNT/β-catenin and JNK signaling pathways.
**Margono et al. (2020)** [47]	DPSCs	hDPSCs + 1%, 5%, and 25% A-PRF conditioned media 1%, and 10% Fetal Bovine Serum as control	TGF-β1 expression in various concentrations of A-PRF in the differentiation process of hDPSCs on day 7	ELISA (TGF-β1 expression)	1%, 5%, and 25% A-PRF, and 10% FBS were not significantly different in producing TGF-β1 (*p* > 0.05), although substantial TGF-β1 expression in 25% A-PRF was higher than that in 1% A-PRF.
**Margono et al. (2023)** [48]	DPSCs	Groups: hDPSCs in Dulbecco’s modified Eagle medium + 10% fetal bovine serum; hDPSCs in 1% A-PRF; hDPSCs in 5% A-PRF; hDPSCs in 10% A-PRF	Migratory speed rate evaluation	Scratch-wound assay at 24 h (migration)	There was a statistically significant difference between the control and 5 and 10% of A-PRF groups (*p* < 0.05), but there was no statistically significant difference between the control and 1% A-PRF group. There was a statistically significant difference between 10% APRF group and 1 and 5% A-PRF groups (*p* < 0.05), but there was no statistically significant difference between 5% A-PRF and 1% A-PRF groups.
**Moreira et al. (2021)** [49]	SCAPs isolated from adult teeth	Chitosan hydrogel was used as a scaffold. The parameters for Photobiomodulation (PBMT) included the use of a continuous-wave indium–gallium–aluminum–phosphide (InGaAlP) diode laser with a wavelength of 660 nm and a spot size of 0.028 cm^2^. The following parameters were applied in contact and punctual irradiation mode: 20 mW, 0.71 W/cm^2^, 3 J/cm^2^ (4 s) or 5 J/cm^2^ (7 s), and 0.08 J or 0.14 J, respectively. Irradiations were performed twice (6 and 12 h after seeding) underneath each well (kept in an incubator under standard culture conditions). Non-irradiated group (positive control); 3 J/cm^2^ for 4 s; 5 J/cm^2^ for 7 s.	Cell migration and viability	MTT assay at 72 and 120 h (viability), and transwell migration assay at 18 and 48 h (migration)	At 72 h, both PBMT groups showed increased cell proliferation compared to the control group. At 120 h, only the PBMT 5 J/cm^2^ group presented a higher number of cells than the control group. At the experimental time of 18 h, both PBMT groups presented increased cell migration through the chitosan scaffold when compared to the control groups. (*p* < 0,05). At 48 h, the highest migration occurred in the PBMT 5 J/cm2 group (*p* < 0.0001).
**Mu et al. (2020)** [50]	hDPSCs and human umbilical vein endothelial cells (HUVECs)	Self-assembling peptide hydrogels RADA16-I used as a scaffold. Groups: DPSC; Stem cell factor (SCF) + DPSC; RADA16-I-2D; SCF-RADA16-I-2D; RADA16-I-3D; SCF-RADA16-I-3D.	Cell viability, adhesion, activity, proliferation, and migration. Angiogenesis on HUVECs and SCF + HUVECs groups. Protein expression.	Transwell migration assay at 24 h (migration), Calcein AM dye at 1 and 3 d (activity), CyQUANT NF reagent at 1, 4, 7 d (proliferation), cytoskeleton staining at 3, 24 h (adhesion), and Western blot assays at 3, 6, 9 h. Calcein AM and photographed again under a fluorescence microscope at 3, 6, 9 h (angiogenesis)	RADA16-I provided a 3D environment for DPSCs and facilitated, in addition to SCF adhesion (*p* < 0.01), migration (*p* < 0.05), and activity (*p* < 0.05). The study demonstrated that the combination of SCF and HUVECs significantly enhanced the formation of vascular-like structures (*p* < 0.01) and release of VEGFA (*p* < 0.01). The proliferation of DPSCs with the SCF was better than that without the SCF. There was a statistical difference (*p* < 0.01) between group 100 ng/mL and groups 0, 25, and 200 ng/mL.
**Noohi et al. (2023)** [51]	Primary SCAPs	Methacrylated chitosan (ChitMA) and methacrylated collagen (ColMA) in different proportions: ChitMA/ColMA 25/75, 50/50, and 75/25 photocross-linked upon exposure to visible light for specified times (20, 30 and 40 s) with or not an extract of platelet-rich fibrin (PRFe)	Cell viability, attachment, spreading, morphology, and gene expression of ALP, COL I, DMP1, DSPP, VEGFA	MTT at 1, 4, 7, 14 and 21 days (viability), Phalloidin/DAPI at 7 days (morphology), live/dead assays (Thermo Fisher Scientific, Waltham, MA, USA) at 45 m after seeding cell (spreading), alizarin red S staining at 21 days (mineralization), hematoxylin-eosin staining at 21 days (histological analysis), RT-PCR analysis at 14 and 21 days (gene expression) and immunohistochemical detection to detect expression of DSPP and COL I at 21 days	The PRFe-loaded hydrogel exhibited higher cytocompatibility to SCAP. PRFe-loaded hydrogel, compared to those without PRFe, significantly promoted cell proliferation at 4 (*p* < 0.05) and 7, 14, 21 d (*p* < 0.0001), spreading and morphology (significant images were taken) and supported the odontogenic differentiation of SCAP in vitro by its promotion of biomineralization and upregulating the gene expression for ALP, COL I (*p* < 0.05), and DMP1 (at 14 *p* < 0.05 and 21 d *p* < 0.01). It facilitated angiogenesis by enhancing (*p* < 0.01) VEGFA gene expression.
**Pan et al. (2024)** [52]	SCAPs from the healthy third molars without apical closure extracted from five volunteers	The concentrations used for the injectable platelet-rich fibrin extract (i-PRFe) and traditional platelet-rich fibrin extract (PRFe) in the study were as follows: 1×, 1/2×, 1/4×, and 1/8×. 1/4× i-PRFe and 1/4× PRFe were selected as the optimal concentrations.	Cell proliferation, migration, mineralization, angiogenesis, and gene expression of ALP, DMP1, DSPP, OCN, angiogenin (ANG), C-X-C motif chemokine receptor 4 (CXCR4), VEGF and platelet-derived growth factor receptor alpha (PDGFRA)	CCK-8 (Sigma-Aldrich, St. Louis, MO, USA) at 1, 3, and 5 days (proliferation), transwell assay at 1 d (migration), alizarin red S staining at 7, 14, 21 days (mineralization), tube formation assay at 7 days (angiogenesis), and RT-qPCR (gene expression)	i-PRF released a higher concentration of growth factors compared to PRF (*p* < 0.05). The i-PRFe group showed a significantly higher cell proliferation rate and migration than the PRFe group (*p* < 0.05) after 1, 3, and 5 d of culture. i-PRFe induced a denser formation of mineralization nodules compared to PRFe after 21 days, with significant (*p* < 0.05) upregulation of odontogenic gene expressions (ALP, DSPP, OCN) in the i-PRFe group. The tube formation assay demonstrated that the area of tubular structures was larger in the i-PRFe group than in the PRFe group (*p* < 0.05).
**Rewthamrongsris et al. (2024)** [53]	SCAPs from Immature third molars	Varying concentrations of simvastatin (100, 250, 500, and 1000 nM)	Cell viability, proliferation, migration, apoptosis, cell cycle analysis, and colony forming unit (CFU)	Live/dead assay (Thermo Fisher Scientific, Waltham, MA, USA) at 24 and 48 h (viability), MTT assay at 1, 3, 7 days (proliferation), flowcytometry analysis (cell cycle), propidium iodide/annexin V staining (apoptosis), Comassie blue staining at 12 days (CFU assay), scratch migration assay at 24 and 48 h (migration)	Simvastatin reduced cell numbers at days 3 and 7. In addition, simvastatin significantly decreased colony formation in both colony number and cell density in a dose-dependent manner. An increase in apoptosis was observed at day 7. There was a statistically significant increase in the sub-G0 population. An in vitro cell migration was attenuated in a dose-dependent manner. All results are statistically significant (*p* < 0.05)
**Ruangsawasdi et al. (2017)** [54]	Primary human mesenchymal stem cells (hMSCs)	Stem cell factor (SCF) at various concentrations (100, 1000, 2500, 5000 ng/mL)	Cell migration, proliferation, and odonto/osteoblasts differentiation	Non-coated m-Slide Chemotaxis 3D assays (migration), WST-1 assay (proliferation), and ALP activity (differentiation)	SCF significantly (*p* < 0,05) directed the migration, enhanced proliferation, and increased ALP activity (14 d) at 100 ng/mL SCF. hMSCs migrated toward a SCF gradient with a significant effect when 2.5 (*p* = 0.0041) and 12.5 (*p* = 0.0021) mg/mL SCF were loaded in one of the reservoirs.
**Shrestha et al. (2019)** [55]	SCAPs	Core-shell nano-system (TD-NS) that released sequentially transforming growth factor-β1 (TGF-β1), reaching a concentration of 2 ng/mL in 7 days to 14 days, which tapers subsequently. Then, dexamethasone (Dex) was released linearly from 9 days to 28 days. The control group is described as the group where SCAPs are cultured without the addition of nanoparticles or bioactive molecules. The case group is the TD-NS group, which involves SCAP treated with the core-shell nano-system (TD-NS) that sequentially releases TGF-β1 and Dex.	Cell viability, morphology, migration, adherence and gene expression of ALP, DSPP, DMP-1 and glyceraldehyde 3-phosphate dehydrogenase (GAPDH)	Cell titer blue cell viability Assay at 1, 3, 7, 14 days, transwell assay at 24 h (migration), calcein AM staining at 24 h (morphology), ALP activity assay at 3, 7, 14 days and ARS staining at 14 days (differentiation), immunofluorescence at 4 h (adherence and detection of DMP-1 and DSPP), and qRT-PCR (gene expression)	Adhesion, cytoskeletal F-actin alignment and average area per cell in the microscopic field of view of the TD-NS-coated surface was significantly higher compared with the uncoated surface (*p* < 0.05). Migration study showed that SCAP migration in the Free-TD group was significantly higher after 5 h than SCAP in the TDNS Group and those after 24 h compared to 5 h. The TD-NS group showed a significantly higher (*p* < 0.05) osteo/odontogenic differentiation compared to the control and free TGF-β1 group (Free-TD).
**Soares et al. (2021)** [56]	Primary hDPCs	The DPCs were maintained in a three-dimensional (3D) culture SV–releasing CH-Ca as a scaffold. Cells were cultured on different scaffold: CH, CH-Ca, CH-SV, CH-Ca-Sv.	Cell viability, metabolism, migration, gene expression of ALP, Col1A1, DSPP, and DMP-1. Scaffolds in vitro biological characterization and APC assay—scaffold surface analysis.	Live/dead assay (Thermo Fisher Scientific, Waltham, MA, USA) at 14 days (viability), Alamar Blue assay (Thermo Fisher Scientific, Waltham, MA, USA) at 1, 7, 14 days (metabolism), F-actin staining at 14 days (cytoplasmic filaments) immunofluorescence to detect dentin sialoprotein, ALP activity assay and Alizarin Red assay at 14 and 21 d respectively (mineralization), and RT-qPCR at 14 d (gene expression)	CH-Ca-SV showed significantly higher cell metabolism at 1 and 7 days (*p* < 0.05), enhanced odontoblastic marker expression (ALP, DSPP, DMP-1) (*p* < 0.05), and greater cell migration in the APC assay (*p* < 0.05) compared to CH, CH-SV, and CH-Ca. In vivo, CH-Ca-SV increased bone volume in rat calvaria defects (*p* < 0.05) and exhibited more mineralization foci (no *p*-value). The scaffold’s sustained release of Ca^2+^ and SV (no *p*-value) promoted cell adhesion, proliferation, and differentiation, making it a promising cell-homing platform for dentin and bone tissue engineering.
**Srisuwan et al. (2022)** [57]	HSCAPs from immature mandibular third molars recruited from 16- to 25-year-old healthy patients	TGFβ1 at 1.25, 2.5, 5, and 10 ng mL^−1^. Root rinsing with different irrigants: normal saline solution (NSS) for 20 min, EDTA for 20 min and Chlorhexidine (CHX) /EDTA (2% CHX 5 min, followed by EDTA for 15 min).	Cell proliferation, mineralization and release of TGF-b1 from root canal dentin	Alamar Blue assay (Thermo Fisher Scientific, Waltham, MA, USA) at 0, 2, 4, 6, 24, 48, 72, 96, 120 h (proliferation), alizarin red assay staining at day 21 (mineralization), ELISA (TGF-b1)	A significant reduction in cell proliferation was observed in all groups containing TGF-b1 from 4 h up to 120 h after contact (*p* < 0.05). When comparing different concentrations, the reduction was apparently observed in groups using higher TGF-b1 (5 and 10 ng mL^−1^) than the lower groups (1.25 and 2.5 ng mL^−1^) (*p* < 0.05). Among the different concentrations tested, the highest amount of calcified matrix was observed in the group treated with TGF-b1 at 1.25 ng mL^−1^ (*p* < 0.05). The study also found that TGF-b1 was released from dentin only EDTA was used as a root canal irrigant (*p* < 0.05).
**Sun et al. (2019)** [58]	HDPSCs from healthy third human molar and LPS- stimulated dental pulp cells	Vascular endothelial growth factor A (VEGFA), PF573228 (inhibitor of FAK), LY294002 (inhibitor of PI3K), SB203580 (inhibitor of p38) and SU5416 (inhibitor of VEGFR2) knockdown the expression of VEGFR1 (Flt-1) and VEGFR2 (Flk-1/KDR)	Cell migration and molecular pathways associated: FAK, PI3K/Akt and MAPK signaling pathways via receptors VEGFR1 or VEGFR2	Immunofluorescence staining; transwell migration stained, wound healing assays and qRT-PCR at 24 h (migration: effects of PF573228, LY294002, SB203580 and SU5416 inhibitors). Western blot analysis (FAK, PI3K, Akt and p38). Silence RNA (siRNA) transfection (VEGFR1 (Flt-1) and VEGFR2 (Flk-1/KDR) expression).	Increased expression of VEGFA level (*p* < 0.05); concentration-dependent migration and dose (10, 20, 50, 100 ng/mL^−1^) and time-dependent FAK, PI3K, Akt and p38 signaling pathways activation promoted by VEGFA. VEGFA-induced migration was significantly inhibited with PF573228, LY294002, SB203580 or SU5416 (*p* < 0.05); signaling pathways VEGFA-activation was significantly suppressed by pre-treatment with SU5416 or transfection with siRNA of VRGFR2 (*p* < 0.05).
**Terranova et al. (2021)** [59]	DPSC from fresh pulp tissue extracted from human third molars	Composite membrane made of electrospun poly(lactic acid) (PLA) nanofibers and electrosprayed polycaprolactone (PCL) with tannic acid (TA). Microparticles coated or not with gelatin (PLA/PCL-TA and G@PLA/PCL-TA).	Cell morphology. Cell viability, proliferation, migration, odontogenic differentiation, and mineralization	SEM at 1, 7 days (cell morphology) LIVE/DEAD assay (Thermo Fisher Scientific, Waltham, MA, USA) at 1 day (viability), Alamar Blue assay (Thermo Fisher Scientific, Waltham, MA, USA) at 1, 3, 7, 10, 14 days (proliferation), ALP activity assay at 7, 14 days (differentiation), calcium colorimetric assay (mineralization), and transmigration assay by confocal laser scanning microscope at 14, 28 days (migration)	Gelatin coating on the membranes improved cell viability after 24 h than scaffold without gelatin (*p* < 0.05). On day 7, the cell number was significantly (*p* < 0.01) comparable between PLA/PCL-TA membranes and TCP compared to G@PLA/PCL-TA membranes. On day 10 and 14, number of cells was significantly (*p* < 0.01) higher on the TCP surface with no difference in proliferating cells between both membranes. At day 7 and 14, cells cultivated in odontogenic differentiation medium on PLA/PCL-TA or G@PLA/PCL-TA membranes had significantly (*p* < 0.05) higher ALP activity than negative control cells and comparable to positive control cells.
**Tian et al. (2020)** [60]	DPSCs from healthy human premolars or impacted third molars	miR-584mimics and miR-584 inhibitor (negative control); pcDNA3.1-TAZ-3′UTR-MUT and pcDNA3.1-TAZ-3′UTR-WT; pcDNA3.1-TAZ and pcDNA3.1-NC; si-TAZ and si-NC.	Analysis of TAZ and miR-584. Cell migration, proliferation and cycle. Luciferase activity.	QRT-PCR and immunoblotting (expression of genes and protein). Immunofluorescence assay of DPSCs at 24 h. Luciferase assays at 48 h, CCK-8 Assay at 3, 5, 7 days post-transfection (proliferation), flow cytometry analysis of cell cycle, Transwell migration assay at 24 h (proliferation).	miR-584 expression levels were upregulated in aging dental pulp tissue, is downregulated in the proliferation of DPSCs and represses proliferation and migration. miR-584 represses the proliferation and migration of DPSCs. TAZ overexpression reverses the inhibition of miR-584 mimics on DPSCs’ proliferation and migration. TAZ promotes the proliferation and migration of DPSCs.
**Wang et al. (2022a)** [61]	hDPSCs from third molars, primary human bone marrow stem cells (hBMSCs), HUVECs	Schwann cell-derived extracellular vesicles (SC-EVs) at various concentrations (0, 100, 200, or 400 μg/mL)	Cell proliferation, migration, osteogenic differentiation, tube and angiogenesis	Cell Counting Kit- 8 assay (Sigma-Aldrich, St. Louis, MO, USA) at 7 d (proliferation), Transwell assays at 12 h (migration), microscopic analysis of HUVECs at 14 h (angiogenesis), Alizarin Red staining at 7 d (differentiation), and immunofluorescent Staining at 3 d (neurite outgrow)	SC-EVs have a positive impact on the proliferation, migration, and differentiation of DPSCs and BMSCs. CCK-8 assay showed that SC-EVs significantly (*p* < 0.05) enhanced the proliferation of DPSCs at a concentration of 200 μg/mL. SC-EVs promoted significant (*p* < 0.05) at 200 and 400 μg/mL tube formation, neurite outgrowth and neuron migration. Representative images of Alizarin Red S staining of DPSCs and DPSCs were taken.
**Wang et al. (2019)** [62]	hDPSC	These cells were cultured and assessed for their biological characteristics when exposed to different scaffolds, including the Human Freeze-dried Dentin Matrix (FDDM), dentin, HA, and coverslips with culture medium supplemented with 10% fetal bovine serum (FBS)	Cell viability, morphology and proliferation. Collagen secretion, ALP activity, COL-1, FN, ALP, and DSPP and protein expression of fibronectin, biglycan, DSP, DM, and BSP.	SEM (morphology), MTT assay at 1, 3, 5 and 7 d(viability), Sirius Red (Sigma-Aldrich) staining (collagen secretion). ALP staining at 7 d (ALP activity), RT-qPCR at 14 d (gene expression), and Immunofluorescence Staining at 17 h (protein expression).	FDDM has mechanical and biochemical and biophysical characteristics similar to those of dentin. DPSCs cultured on FDDM and dentin exhibited superior viability and proliferation than that in HA liquid extract (*p* < 0.05 for 48 h). The study found that DPSCs cultured on FDDM showed increased collagen secretion and ALP activity but a decreased mineral capability compared with DPSCs cultured with PBS or HA. Generally, DPSCs cultured on FDDM and dentin expressed the highest levels of fibronectin and COL-1, whereas cells cultured on HA expressed the lowest levels.
**Wang et al. (2021a)** [63]	hPSC	0.1 or 0.5 μM Simvastatin or LY294002 transfection	Expression levels of AKT, miR-9 and KLF5, or determine the effect of miR-9, cell proliferation, and luciferase	Cell Counting Kit- 8 assay (Sigma-Aldrich, St. Louis, MO, USA) (proliferation), Western blot analysis and qRT-PCR (gene expression), luciferase assay	Simvastatin enhances DPSC proliferation by upregulating PI3K and p-AKT, downregulating miR-9, and increasing KLF5 expression, with LY294002, miR-9, and KLF5/AKT siRNAs counteracting these effects (*p* < 0.05 where reported).
**Wang et al. (2022b)** [64]	SCAPs from extracted third-molar teeth	Protein arginine methyltransferase 6 transfection (PRMT6)	Cell Proliferation and migration, osteo/odontogenic differentiation, and expression of HA and PRMT6	CellTrace CFSE Cell Proliferation Kit, ALP activity, ARS staining at 2 w (differentiation), Western blot (protein expression), RT-PCR, scratch assay, and Transwell assay at 24, 48 h (migration)	Knockdown of PRMT6 promoted the osteo/odontogenic differentiation compared with the control group, as detected by ALP and ARS and migration ability. Overexpression of PRMT6 promoted contrary effects.
**Wang S. et al. (2020)** [65]	hBMSCs	Cells were cocultured with nanobioactive glass (nBG) at a concentration of 0.5 mg/mL. The nBG was applied to dentin discs	Cell-biomaterial interaction, cell morphology, migration, adhesion	IncuCyte S3 live cell imaging system (Essen BioScience, Ann Arbor, MI) every 72 h (interaction), confocal microscopy at 2 h and 3 d (morphology and adhesion), Transwell migration assay (migration)	BMSCs exhibited better initial attachment to nBG-coated dentin surfaces and significantly (*p* < 0.05) greater migration compared to untreated dentin surfaces. The morphology of BMSCs on nBG-coated surfaces transitioned from a rounded phenotype to a spread, polygonal shape. The nBG particles demonstrated good biocompatibility.
**Wang et al. (2021b)** [66]	Primary hBMSCs	Dentin-derived TGFβ1 release and activation by pH 7.4 or pH 10 injectable hydrogel (MS-Gel)	Cell migration, and viability, accumulation of phospho-Smad 2/3 in cells, and protein (phospho-Smad 2/3, Smad 2/3, Smad 1/5/9, phospho Smad 1/5/8, anti-COL-1, RUNX2), GAPDH and gene (COL-1, DSPP, BSP and OSX) expression	Transwell migration essay at 3 d (migration), Cell Counting Kit-8 (CCK-8) (Sigma-Aldrich, St. Louis, MO, USA) at 1, 3, 5 d (viability), immunofluorescence (accumulation), Western blotting (protein expression), and qRT-PCR (gene expression)	Alkaline MS-Gel activated dentin-derived TGFβ1 for cell migration. hBMSCs proliferated effectively in the presence of MS-Gel, without negative effects on cell viability. Gene expression analysis revealed a significant increase in osteogenic markers, suggesting a promotion of cellular differentiation. Results were considered statistically significant (*p* ≤ 0.05; *p* ≤ 0.01; *p* ≤ 0.001).
**Wang et al. (2023)** [67]	hDPSC from human premolars and HUVECs	The hDPSCs were cultured within these hydrogels: hydroxypropyl chitin (HPCH), HPCH/CW (chitin whisker) and HPCH/CW/Exo (exosomes isolated from hDPSC)	Cell viability, proliferation morphology, migration, odontogenesis and angiogenesis differentiation, and gene expression of OCN, DSPP, COL1a, Runx2	CCK-8 at 1, 3, 5 d (viability on cells co-cultured with the extract of hydrogels or encapsulated within the hydrogels), Live/dead staining (Thermo Fisher Scientific, Waltham, MA, USA) at 1, 3 d (proliferation), confocal laser scanning microscope (morphology), scratch-wound healing assay at 0 and 24 h and tube formation essay at 12 h (angiogenesis), ARS at 21 d, Quantitative ALP assay at 14 d (differentiation), RT-PCR (gene expression)	Compared with the pure HPCH hydrogel, the cell proliferation was significant (*p* < 0.05) at day 7 in both HPCH/CW and HPCH/CW/Exo encapsulating cells groups. Odontogenic differentiation was demonstrated by ALP and ARS staining, showing that the hDPSCs encapsulated in the HPCH/CW/Exo hydrogel had a higher (*p* < 0.01 for quantitative analysis of ALP) positive area than the other groups after 14 and 21 days of co-culture, respectively. The gene expression of OCN, DSPP, COL1a, and Runx2 of cells cultivated on HPCH/CW/Exo is significantly (*p* < 0.05) higher at 14 d. Quantitative analysis showed that the number of nodes and total tube length from the HPCH/CW/Exo group was higher (*p* < 0.05) than that of the HPCH/CW group.
**Wei et al. (2021)** [68]	SCAPs rom normal human impacted third molars	Silk fibroin (SF), SF-RGD, SF-RGD-stem cell factor (SF-RGD-SCF) scaffold	Cell migration, proliferation, adhesion, and growth status	Transwell Migration Assay at 24 h (migration), digestion counting method at 1 and 7 d and live/Dead assay (Thermo Fisher Scientific, Waltham, MA, USA) at 7 d (proliferation), immunofluorescence at 1, 7 d, and laser confocal microscopy (adhesion and growth status)	Cells on the SF-RGD-SCF scaffold proliferated significantly (*p* < 0.05) more at 1 and 7 d and migrate more than those on the other scaffolds after 7 d seeding (*p* < 0.01). Live/dead cell staining results showed that almost all the adhered cells were alive after 7 d. Furthermore, cell counting showed that the number of cells on the SF-RGD-SCF scaffold was highest after both 1 and 7 d (*p* < 0.05).
**Widbiller et al. (2022)** [9]	36 single-rooted, caries-free and unrestored human teeth	Concentration of TGF-b1 after centrifugation with cooling centrifuge at 21 °C and 14,000× *g* filters with a molecular weight cut-off of 3000 Da and 10,000 Da after 3, 6, 9, 12, 15, and 18 m of 500 µL of solution collected from EDTA of final irrigation in molars treated. Different types of treatment: (1) Irrigation with 2% sodium hypochlorite, 0.2% chlorhexidine, and 0.9% saline. (2) Root canals enlarged with RECIPROC blue R50 (size 50, taper 0.05) from RECIPROC blue R25 (25, 0.08 taper). (3) Intracanal dressing of calcium hydroxide for 72 h.	Concentration and absolute mass of soluble TGF- 1 in EDTA collected from root canals after ultrasonic activation	Solid-phase sandwich ELISA for TGF- 1	Irrigation with sodium hypochlorite reduced the release of TGF- 1 from root dentin significantly compared to controls with saline and chlorhexidine (*p* < 0.0160), but the use of sodium hypochlorite for irrigation after the canal enlargement and subsequent use of calcium hydroxide facilitated a significant increase in concentrations and masses of TGF- 1 (*p* < 0.0397).
**Xiao et al. (2019)** [69]	SCAPs from disease-free impacted third molars at the stage of root development	Synergistic effects of stromal cell-derived factor-1α (SDF-1α) and bone morphogenetic protein-2 (BMP-2) on the odontogenic differentiation of SCAP cultured in the VitroGel 3D system, an animal origin-free polysaccharide hydrogel.	Cell morphology, viability, proliferation, odontogenic differentiation genes (ALP, Runx-2, BSP, DMP-1, DSPP and OCN) and proteins (Runx-2, DMP-1, DSPP, BSP, OCN, GAPDH)	SEM at 1, 4, 7 days (morphology), live and dead cell staining at 4 days (viability) and CCK-8 assays at 0, 1, 4 and 7 (proliferation), ALP activity at 3, 7, 11 and 14 days, Alizarin Red S or 2% Oil red O at 4 w(differentiation), RT-PCR at 3, 7 and 14 d(gene expression), Western blot analysis (protein expression)	The SCAP cultured in the VitroGel 3D hydrogel system demonstrated favorable viability and proliferation. Cell proliferation assays revealed that cells proliferated faster at days 1, 4 and 7 than that at day 0 in both 3D hydrogel culture and 2D culture (*p* < 0.05). Real-time RT-PCR and Western blot analyses indicated that the expression of key genes ALP, Runx-2, BSP, DMP-1 and OCN genes was upregulated in the presence of both SDF-1α and BMP-2 compared to their control groups at days 3, 7 and 14 (*p* < 0.05) whereas the difference in expression levels of the DSPP gene between these groups was only significant at days 7 and 14 (*p* < 0.05).
**Yuan et al. (2023)** [70]	DPSCs and HUVECs	This study utilized porcine dental pulp to derive the decellularized extracellular matrix (dECM). 5, 7.5, 10 mg/mL dECM, GelMA hydrogel and PBS as Blank	Cell viability, proliferation, migration, tube formation, odontogenic, and neurogenic differentiation by expression of Nestin, GFAP, and DMP-1	CCK-8 analysis at 1, 4, and 7 days (viability), Transwell migration assay with SEM at 6 h (migration), tube formation and RT-PCR (gene expression)	dECM hydrogels demonstrate favorable biocompatibility and chemotactic activity, facilitating cell survival, proliferation, and migration. dECM Hydrogels Exhibit Strong Angiogenic Properties and Facilitate DPSCs’ differentiation into odontogenesis.
**Zhang et al. (2020)** [71]	hDPSCs from extracted sound third molars from human	Injectable hybrid RGD-alginate/0.5% laponite (RGD-Alg/0.5%Lap) hydrogel microspheres, co-encapsulating hDPSCs and vascular endothelial growth factor VEGF	Cell viability. Cellular morphology. Extracellular matrix proteins’ deposition. Odontogenic differentiation genes Col- I, DMP-1, and ALP	Live/Dead assay (Thermo Fisher Scientific, Waltham, MA, USA) at 1, 4, 7, 14 (viability), staining of F-actin and cell nuclei (morphology), immunofluorescent staining of extracellular matrix (ECM) at 7 days, and RT-PCR at 1, 3, 7 days (gene expression)	The cell viability rates exceeded 90% in day 1, 4, 7, and 14 in RGD-Alg microspheres. Microspheres de- posited a considerable amount of ECM, rich in laminin, fibronectin, and integrin β1 after being cultured for 7 days. Also, the four tested odontogenic-related genes expression in all groups exhibited a gradual increase trend on the whole and peaked at day 7.
**Zhao et al. (2023)** [72]	HDPCs isolated from extracted third molars	HDPCs were cultured on dentine slices with or without phase-transited lysozyme (PTL)	Cell adhesion, migration, morphology, and proliferation. Odontogenic differentiation. Expression levels of specific genes DSPP, BSP, and osteopontin.	DiI staining (to label living human dental pulp cells to assess their adhesion to dentine slices, with or without PTL coating, scanning electromicroscopy at 3 days (morphology), MTT assay at 1, 2, 3 days (viability), Transwell migration assay at 1, 2 days (migration), RT-qPCR at 3, 7, a14 days (gene expression), Western blot at 7, 14 days, and ARS staining 14, 21 days (mineralization)	PTL showed no negative effect on cell cycle of HDPCs and compared with the blank group, HDPCs labeled with DiI staining showed significantly more adhered cells at 48 h (*p* < 0.05), extending cell processes and more finger-like or reticular pseudopodia on PTL-coated dentine slices. The results of MTT and Transwell assay showed that PTL promoted the proliferation (*p* < 0.05) and migration (*p* < 0.01) of HDPCs, respectively. Compared with the blank group, the gene expression of DSPP, osteopontin and bone sialoprotein in HDPCs cultured on PTL was significantly upregulated on day 3 and 7 (*p* < 0.05), while the protein expression of DSPP showed no significant change on both day 7 and day 14. ARS showed that PTL promoted more mineralization nodules formation of HDPCs (*p* < 0.05).
**Zheng et al. (2023)** [73]	hDPSCs	Cells were cultured, functionalized gelatin methacrylate (GelMA) microspheres that were modified with a soluble decellularized dental pulp matrix (dpECM@GM)	Physical characterization. Odontogenic, angiogenic and neurogenic differentiation. Gene expression.	In Vitro Tube Formation Assays. Immunofluorescence staining. qPCR.	dpECM@GM as a type of bioscaffold exhibited good cytocompatibility, which could support cell proliferation. hDPSCs cultured on dpECM@GM showed significantly higher expression levels of odontogenic markers, such as DSPP and DMP-1, angiogenic markers and neuron-specific genes compared to those cultured on GM and standard culture plates.
**Zhu et al. (2024)** [74]	hDPSCs. OriCell^®^ human stem cell osteogenesis differentiation-inducing medium was used for cell culture for 7 days.	Effects of programmed cell death ligand 1 (PD-L1) on cell infecting these with PD-L1 overexpression lentiviral vector (LV-PD-L1) and CTCF suppression lentiviral vector (sh-CTCF)	Cell viability, osteogenic differentiation. Expression of glyceraldehyde-3-phosphate dehydrogenase (GAPDH), PD-L1, PD-1, CTCF, OCN, ALP, COL1α1, DSPP, DMP-1 and Runx2.	CCK-8 assay (viability), ALP activity and ARS staining (differentiation), Western blotting, RT-qPCR (gene expression)	PD-L1 overexpression was found to inhibit lipopolysaccharide (LPS) and induced pro-inflammatory cytokine upregulation. The overexpression of PD-L1 significantly enhances the proliferation and osteo/odontogenic differentiation of hDPSCs.

**Table 3:** In vivo studies selected.

Author/s and Year	Populations	CF-RET Intervention	Biological Effects	Assay Used and Conditions	Principal Findings
**Abo-Heikal et al. (2024)** [75]	23 patients classified as ASA class I physical status (normal healthy patient) aged between 9 to 24 years had a total of 24 immature traumatized necrotic maxillary anterior teeth. No sex predilection. The included teeth had an apical opening greater than 1 mm measured labio–palatally or mesiodistally on the axial plane.	Working length was determined. Minimal instrumentation on the canal walls and copious irrigation (20 mL) with 1.5% NaOCl for 5 min, 1.5 mm short of the working length using a gauge 25 side-vented irrigation needle. The canal was then irrigated with 20 mL of saline for 5 min as a final flush, dried with sterile paper points and filled with ultracal injectable calcium hydroxide (Ca(OH)_2_). An additional disinfection visit was planned only if any signs of persistent infections were noticed. After 2 weeks, patients were randomly assigned to receive either i-PRF (Group 1) or PRP (Group 2).	Pulp sensitivity if the tooth in question gave a response (even at high current). Root length and canal diameters were calculated at three canal levels (coronal, middle, and apical).	Assessment at 6 and 12 months post-treatment. Electric pulp tester (sensitivity) and 3D cone-beam computed tomography (CBCT) 6 cm height × 6 cm Diameter. X-ray tube voltage: 89–90 KVp, X-ray tube current: 6–8 mA and a voxel size of 85 microns (root length and canal diameters).	Both i-PRF and PRP demonstrated positive regenerative effects, but i-PRF was highlighted as exhibiting a significantly (*p* = 0.008) superior performance in reducing apical canal diameter. Similar outcomes were observed for pulp sensitivity restoration in both groups, with no significant difference in response to the electric pulp tester.
**Shetty et al. (2021)** [76]	A total of 50 teeth from 42 patients (29 males and 13 females) aged 9 to 38 years with necrotic pulp and periradicular pathology. The cases presented with either carious pulp exposure (n = 3) or trauma (n = 47). The teeth did not respond to pulp sensibility tests with cold (ice stick) and electric pulp testing.	Regenerative endodontic procedure (REP) in two to three appointments. Disinfection protocol: irrigation with 20 mL of 1.5% NaOCl, 1.0 mg/mL triple antibiotic paste (1:1:1 ciprofloxacin: metronidazole:minocycline with distilled water). After resolution of clinical signs and/or symptoms: irrigation with 20 mL of 1.5% NaOCl and subsequently with 5 mL of 17% ethylenediaminetetraacetic acid (EDTA). Bleeding was induced into the coronal portion of the root canal space by laceration of the apical tissue with a sterile 23-gauge needle. A sterile biodegradable bovine collagen plug (CollaPlug, Zimmer Biomet Holdings Inc., Warsaw, USA) was placed on the blood clot to serve as a scaffold. A coronal seal of either white mineral trioxide aggregate (ProRoot; Dentsply Sirona, Ballaigues, Switzerland) or Biodentine (Septodont, France) was placed, following which the tooth was sealed with a glass ionomer cement.	Root length and canal diameters were calculated at three canal levels (coronal, middle, and apical). The cone-beam computed tomography periapical index (CBCTPAI) was used to evaluate periradicular lesions. Clinical tests were performed to assess pulp sensibility, spontaneous pain, pain on palpation, sinus tract, swelling and crown discoloration.	Assessment at 6, 12 and 18 months post-treatment. 48 months clinical follow-up. Clinical test: 3D CBCT (periradicular lesion, root length and canal diameters)	The study reported a high success rate of 94.6% for REP, defined by clinical functionality and radiographic evidence of continued root development after 48 months’ follow-up. Significant (*p* < 0.05) increase in root length, decrease in pulp space diameter, and periradicular radiolucency.
**Theekakul et al. (2024)** [77]	120 necrotic immature permanent teeth with/without periapical lesions from 108 patients (55% females) treated with REPs. 63.3% of the patients were younger than 12 years old. All teeth had either negative electronic pulp test (EPT) or cold test results. Teeth with irreversible pulpitis, in which bleeding could not be stopped, and vital pulp therapy could not be performed, were also included.	(1) Initial cleaning and disinfection of the root canal using 20 mL/canal 1.25–5.25% NaOCl and normal saline irrigation. Passive ultrasonic irrigation was used for 1 min/canal. (2) Application of intracanal medication (either calcium hydroxide or a full-strength triple antibiotic paste). The intracanal medication period was at least 3–4 weeks. (3) For asymptomatic teeth, REPs were performed: inducing bleeding into the canal to facilitate blood clot formation, followed by placement of a collagen matrix and a calcium-silicate-based capping material (like MTA or Biodentine). (4) The coronal access was lined with glass-ionomer cement (Vitrebond; 3M ESPE, St. Paul, MN) and restored with resin composite (Filtek Z350 XT; 3M ESPE, St. Paul, MN).	Functional retention. Apical diameter, root length, root width (measured at the root levels 50%, 66%, and 80% from the CEJ of the preoperative root length and averaged), and root resorption area (RRA): all root area minus pulp space (Flake’s method). Sensitivity	The follow-up period for the study ranged from 12 to 148 months, with a median of 30.5 months and a mean of 41.7 months. Retention of teeth in the dental arch without clinical signs and symptoms (functional retention) regardless of periapical radiolucency status. Periapical radiograph. Cold test and/or EPT (sensitivity).	The functional retention rate of the treated teeth was 97.5% and 80%; they were categorized as healed. Significant changes were observed in root development parameters post-treatment: - Change in apical diameter: 56.8%. - Change in root length: 8.3%. - Change in root width: 23.2%. - Change in radiographic root area (RRA): 21.7%. The rate of positive response to the sensibility test was 41.7%, with positive correlations found with the use of EDTA irrigation and capping material level above the CEJ (*p* < 0.05). Patients aged >12 years with stage 3 root formation demonstrated a significantly higher risk for disease compared with the patients aged 12 years (*p* < 0.05). An etiology of pulpal disease from dental caries or anomalies significantly contributed to a greater change in the apical diameter compared with that from dental trauma (*p* < 0.05).
**Widbiller et al. (2022)** [9]	38 teeth scheduled for routine endodontic treatment. Various case-dependent parameters were documented, among them tooth type, clinical situation (irreversible pulpitis, pulp necrosis, retreatment), patient age (<40, 40 to 60, >60), and extent of coronal restoration (none, ≤50%, >50% area of access cavity).	Standardized procedures were followed for root canal preparation: root canal preparation to 35, 0.04 under copious irrigation with 2% NaOCl was carried out, intracanal dressing was placed, and the tooth was sealed temporarily. In the second appointment (at least two weeks and not longer than four weeks), irrigation with 2% NaOCl was administered. Canals were dried and filled with EDTA activated by an ultrasonic file for 30 s. EDTA was refreshed and left inside the canal for 10 min, then removed with a carpule syringe and sterile cartridge.	Concentration of TGF-β1.	Prepared solution (500 µL) was loaded into centrifugal filters with molecular weight cut-off of 3000 Da and 10.000 Da and subjected to centrifugation at 14,000× *g* at 21 °C. Volume of EDTA and concentration of TGF-β1 were measured at various time intervals (3, 6, 9, 12, 15, and 18 min).	The amount of TGF-1 collected was significant (*p* < 0.0001) higher in EDTA than in NaCl. Factors such as patient age, clinical situation, type of tooth, and extent of coronal restoration influence the amount of growth factor released. The available amount of TGF-β1 was higher in vital and retreatment cases compared to necrotic cases.
**Zhang et al. (2024)** [78]	56 teeth from 56 healthy patients (Category: American Society of Anesthesiologists class 1) of both genders, aged between 7 and 16 years. Teeth included: (1) mandibular premolars with an immature root apex (apical opening > 1 mm); (2) mandibular premolars not requiring a post or core for the final restoration; (3) traumatically or cariously exposed mandibular premolars; (4) non-vital permanent anterior teeth with or without apical periodontitis.	Both groups underwent similar initial procedures, including access opening, minimal mechanical instrumentation using an ISO #60 H-file, irrigation with 20 mL of 2.5% NaOCl, determination of the working length radiographically, and medication of triple antibiotic paste. The key difference was the application of CGF as a scaffold in the CGF group, while the BLC group relied on a blood clot for the regenerative process for the regenerative endodontic procedure. CGF was obtained from centrifugation (30 s of acceleration, 2 min at 2700 rpm, 4 min at 2400 rpm, 4 min at 2700 rpm, 3 min at 3000 rpm, and 36 s of deceleration) of 10 mL of venous blood. CGF was sectioned into pieces and packed into the root canal, ensuring that it extended approximately 3–4 mm apical to the cementoenamel junction (CEJ).	Clinical presentation (no sinus tract, swelling, and the absence of spontaneous, palpation or percussion pain). Post-operative pain. Changes in root length (RL), apical foramen width (AFW), and root resorption area (RRA): all root area minus pulp space.	Assessment at 1 w, 6, and 12 months post-treatment. Clinical evaluations. visual analog scale at 1 week (pain). Periapical X-rays.	A statistically significant (*p* = 0.005) difference was observed between the BLC group and CGF group at the 12-month follow-up point in RL, and an increase in RRA percent at 6 months.

## Data Availability

The original contributions presented in this study are included in the article/Supplementary Materials. Further inquiries can be directed to the corresponding author.

## Supplementary Materials

The following supporting information can be downloaded at https://www.mdpi.com/article/10.3390/medicina62020375/s1, Table S1: Quality Assessment Score.
