# Supplementary material for: Pulp–Dentin Regeneration via Cell Homing: Current Evidence and Perspectives on Cell-Free Regenerative Endodontic Therapy
Source: Medicina (Kaunas). 2026 Feb 13;62(2):375. doi: 10.3390/medicina62020375 (PMC12942805; doi:10.3390/medicina62020375)
Supplement: Supplementary file 1 [file medicina-62-00375-s001.zip › medicina-4113145-supplementary.pdf]

Supplementary Materials

Table S1: Quality Assessment Score.

	Item 1	Item 2	Item 3	Item 4	Item 5	Item 6	Item 7	Item 8	Item 9	Item 10	Item 11	Item 12	Item 13	Final Score
**Abdelgawad L. M. et al. (2021)**	2	3	3	3	1	2	3	3	3	1	3	N/A	2	29/36
**Al-Ateeq R. et al. (2024)**	3	2	3	2	3	2	3	2	3	1	2	N/A	1	27/36
**Bagio D. A. et al. (2021)**	3	3	3	3	3	2	3	1	3	0	3	N/A	1	28/36
**Bagio D. A. et al. (2024)**	3	3	3	3	2	1	3	2	2	2	3	N/A	2	29/36
**Bagio D. A. et al. (2023)**	3	3	3	3	3	2	3	3	3	2	3	N/A	3	34/36
**Bordini E.A.F. et al. (2022)**	3	3	3	3	3	3	3	3	1	2	3	N/A	2	32/36
**Bordini E.A.F. et al. (2024)**	3	3	3	3	1	1	3	2	3	2	3	N/A	1	28/36
**Caballero-Flores H. et al. (2021)**	3	3	2	3	2	1	2	1	3	1	3	N/A	2	26/36
**Chang B. et al. (2020)**	3	2	2	3	1	2	3	2	3	1	3	N/A	1	26/36
**Chang M. C. et al. (2020)**	3	3	3	3	3	1	3	2	3	1	3	N/A	2	30/36
**Chen Y. et al. (2022)**	3	3	2	3	2	1	2	2	3	1	3	N/A	2	27/36
**Ha M. et al. (2020)**	3	3	3	2	2	1	3	2	2	1	2	N/A	2	26/36
**Hong S. et al. (2018)**	3	2	2	3	2	1	3	1	2	2	2	N/A	1	24/36
**Hong S. et al. (2019)**	3	2	2	3	2	1	3	3	3	1	2	N/A	2	27/36
**Jiang L. et al. (2020)**	3	2	3	3	2	1	3	1	2	2	3	N/A	2	27/36
**Jin W. et al. (2023)**	3	3	3	3	2	2	1	3	2	1	2	N/A	2	27/36
**Karkehabadi H. et al. (2023)**	3	3	3	3	3	1	3	2	2	2	3	N/A	2	30/36
**Ke Z. et al. (2019)**	2	2	3	3	2	1	1	1	2	1	2	N/A	2	22/36
**Kim M. S. et al. (2022)**	3	3	1	3	1	0	2	1	1	0	2	N/A	1	18/36
**Kornsuthisopon C. et al. (2022)**	2	2	3	3	1	1	3	2	3	2	3	N/A	1	26/36
**Lay S. (2023)**	3	3	2	3	1	1	1	2	0	1	2	N/A	1	20/36
**Le S. H. et al. (2024)**	1	2	2	3	1	2	2	2	1	2	3	N/A	3	24/36

**Leite M. L. et al. (2021)**	3	3	3	3	3	2	3	3	3	2	3	N/A	2	33/36
**Leite M. L. et Al. (2022)**	3	3	3	3	3	2	3	3	3	3	3	N/A	2	34/36
**Li M. et al. (2018)**	3	2	2	3	0	2	3	3	3	1	3	N/A	1	26/36
**Liang H. et al. (2023)**	3	2	2	3	0	1	2	1	2	0	2	N/A	2	20/36
**Liu S. et al. (2022)**	3	3	2	3	0	1	3	2	3	1	3	N/A	2	26/36
**Liu Y. et al. (2022)**	3	3	2	3	1	1	3	2	3	2	3	N/A	2	28/36
**Loukelis K. et al. (2023)**	3	3	2	3	1	2	3	3	3	2	2	N/A	3	30/36
**Lv H. et al.**	3	3	3	3	1	1	3	1	3	2	3	N/A	2	28/36
**Margono A. et al. (2020)**	2	3	2	3	1	1	2	1	2	0	2	N/A	1	20/36
**Margono A. et al. (2023)**	3	2	3	3	2	2	3	3	2	2	2	N/A	3	30/36
**Moreira M. S. et al. (2021)**	3	2	3	3	2	1	3	3	2	1	3	N/A	2	28/36
**Mu X. et al. (2020)**	3	3	3	3	2	1	3	2	2	2	3	N/A	2	29/36
**Noohi P. et al. (2023)**	3	3	3	3	2	2	3	3	3	2	3	N/A	3	33/36
**Pan J. et al. (2024)**	3	2	3	3	3	1	3	3	3	1	3	N/A	3	31/36
**Rewthamrongsris P. (2024)**	2	2	3	3	3	1	3	2	2	1	3	N/A	2	27/36
**Ruangsawasdi N. et al. (2017)**	3	2	2	3	2	1	2	2	1	2	3	N/A	3	26/36
**Shrestha S. et al. (2019)**	3	3	3	3	2	2	3	3	1	3	3	N/A	2	31/36
**Soares D.G. et al. (2021)**	3	2	3	3	1	1	3	1	3	2	3	N/A	2	27/36
**Srisuwan T. et al. (2022)**	2	3	3	3	2	1	3	2	3	3	3	N/A	3	31/36
**Sun X. et al. (2019)**	3	3	3	3	2	2	3	2	2	2	3	N/A	3	31/36
**Terranova L. et al. (2021)**	3	2	3	3	2	2	3	2	2	2	3	N/A	3	30/36
**Tian S. et al. (2020)**	3	2	2	3	2	2	3	2	3	2	3	N/A	2	29/36
**Wang D. et al. (2021)**	3	2	3	3	2	3	3	3	3	2	3	N/A	3	33/36
**Wang F. et al. (2019)**	2	3	3	3	2	2	3	3	2	3	3	N/A	3	32/36
**Wang J.-H. et al.**	3	2	3	3	1	1	3	2	2	2	3	N/A	1	26/36
**Wang N. et al. (2022)**	3	2	3	3	1	2	3	1	1	2	3	N/A	2	26/36

**Wang S. et al. (2019)**	2	3	3	3	1	2	2	2	3	2	3	N/A	2	28/36
**Wang S. et al. (2021)**	3	3	3	3	3	3	3	3	3	3	3	N/A	2	35/36
**Wang S. et al. (2023)**	3	2	3	3	2	1	3	3	3	3	3	N/A	3	32/36
**Wei J. et al. (2021)**	3	2	2	3	2	2	3	2	3	2	3	N/A	1	28/36
**Widbiller M. et al. (2022)**	3	3	3	3	3	3	3	3	3	3	3	N/A	3	36/36
**Xiao M. et al. (2022)**	3	3	3	3	3	1	3	3	3	2	3	N/A	3	33/36
**Yuan S. et al. (2023)**	3	3	2	3	2	1	3	3	3	2	3	N/A	3	31/36
**Zhang R. et al. (2020)**	3	3	2	3	3	1	3	2	3	2	3	N/A	3	31/36
**Zhao J. et al. (2023)**	3	2	3	3	2	1	3	2	3	3	3	N/A	3	31/36
**Zheng L. et al. (2022)**	3	3	3	3	1	1	3	1	1	2	3	N/A	3	27/36
**Zhu Y. et al. (2024)**	2	2	2	3	3	1	3	3	3	2	3	N/A	2	29/36
**Abo-Heikal MM. et al. (2024)**	3	3	3	3	3	3	3	3	3	3	3	N/A	3	36/36
**Shetty H. et al. (2021)**	2	3	3	3	3	3	3	3	3	3	3	N/A	3	35/36
**Theekakul C. (2024)**	2	2	3	3	3	3	3	3	3	3	3	N/A	3	34/36
**Widbiller M. et al. (2022)**	3	3	3	3	3	3	3	3	3	3	3	N/A	3	36/36
**Zhang Y. et al. (2024)**	3	3	3	2	3	2	3	3	3	3	3	N/A	3	34/36
